# Berberine treats atherosclerosis via a vitamine-like effect down-regulating Choline-TMA-TMAO production pathway in gut microbiota

**DOI:** 10.1038/s41392-022-01027-6

**Published:** 2022-07-07

**Authors:** Shu-Rong Ma, Qian Tong, Yuan Lin, Li-Bin Pan, Jie Fu, Ran Peng, Xian-Feng Zhang, Zhen-Xiong Zhao, Yang Li, Jin-Bo Yu, Lin Cong, Pei Han, Zheng-Wei Zhang, Hang Yu, Yan Wang, Jian-Dong Jiang

**Affiliations:** 1 State Key Laboratory of Bioactive Substance and Function of Natural Medicines, Institute of Materia Medica, Chinese Academy of Medical Sciences/Peking Union Medical College, Beijing, China; 2 The First Hospital of Jilin University, Changchun, China

**Keywords:** Drug discovery, Biotechnology

## Abstract

Trimethylamine-*N*-oxide (TMAO) derived from the gut microbiota is an atherogenic metabolite. This study investigates whether or not berberine (BBR) could reduce TMAO production in the gut microbiota and treat atherosclerosis. Effects of BBR on TMAO production in the gut microbiota, as well as on plaque development in atherosclerosis were investigated in the culture of animal intestinal bacterial, HFD-fed animals and atherosclerotic patients, respectively. We found that oral BBR in animals lowers TMAO biosynthesis in intestine through interacting with the enzyme/co-enzyme of choline-trimethylamine lyase (CutC) and flavin-containing monooxygenase (FMO) in the gut microbiota. This action was performed by BBR’s metabolite dihydroberberine (a reductive BBR by nitroreductase in the gut microbiota), via a vitamine-like effect down-regulating Choline-TMA-TMAO production pathway. Oral BBR decreased TMAO production in animal intestine, lowered blood TMAO and interrupted plaque formation in blood vessels in the HFD-fed hamsters. Moreover, 21 patients with atherosclerosis exhibited the average decrease of plaque score by 3.2% after oral BBR (0.5 g, bid) for 4 months (*P < 0.05, *n* = 21); whereas the plaque score in patients treated with rosuvastatin plus aspirin, or clopidogrel sulfate or ticagrelor (4 months, *n* = 12) increased by 1.9%. TMA and TMAO in patients decreased by 38 and 29% in faeces (*P < 0.05; *P < 0.05), and 37 and 35% in plasma (***P < 0.001; *P < 0.05), after 4 months on BBR. BBR might treat atherosclerotic plaque at least partially through decreasing TMAO in a mode of action similar to that of vitamins.

## Introduction

Cardiovascular diseases (CVDs) have become the leading cause of mortality with a high rate of death.^1,2^ Among the pathological changes of the diseases, atherosclerosis (AS) is often a common basis of CVDs and an intractable lesion with increased incidences over the years.^3,4^ There are multiple risk factors and molecular mechanisms that contribute to the pathogenesis of AS,^5^ such as high blood lipids or glucose, local or systemic inflammatory responses et al.^1,6^ Recently, accumulating evidences revealed that the gut microbiota is an influential factor in the development and aggravation of AS.^7,8^ For example, metabolites from gut microbiota (such as SCFAs) could play a vital role in down-regulating blood cholesterol or glucose^9,10^ and inhibiting inflammation.^11^ In fact, discovery of drugs that treat diseases through the gut microbiota is becoming attractive.^6,9,12,13^

Trimethylamine (TMA) is an intestinal bacteria-derived metabolite, generated through decomposition of dietary phosphatidylcholine/choline, or L-carnitine, or betaine^7,14–17^ in ingested red meat or animal pluck.^18^ Intestinal TMA absorbed into the blood is further transformed into its proatherogenic form, namely, trimethylamine-*N*-oxide (TMAO) by flavin monooxygenase family members, such as flavin monooxygenase 3 (FMO_3_) in the liver.^19^ Recent study has shown that TMAO is an independent predictor and promoter of AS, different from traditional risk factors.^14^ In fact, TMAO aggravated AS through various mechanisms,^7^ including enhanced effects on foam cell formation,^20^ platelet hyper-reactivity and thrombosis risks,^17,21^ direct activation of the inflammatory response^22,23^ and interfering with the reverse transport of cholesterol.^24^ Intervention studies focusing on TMAO production showed an obviously attenuated effect on AS in animals and human, suggesting the potential of this new target in the gut microbiota.^14,16^ Thus, TMAO is the focused molecule of our study.

**Fig. 1:**
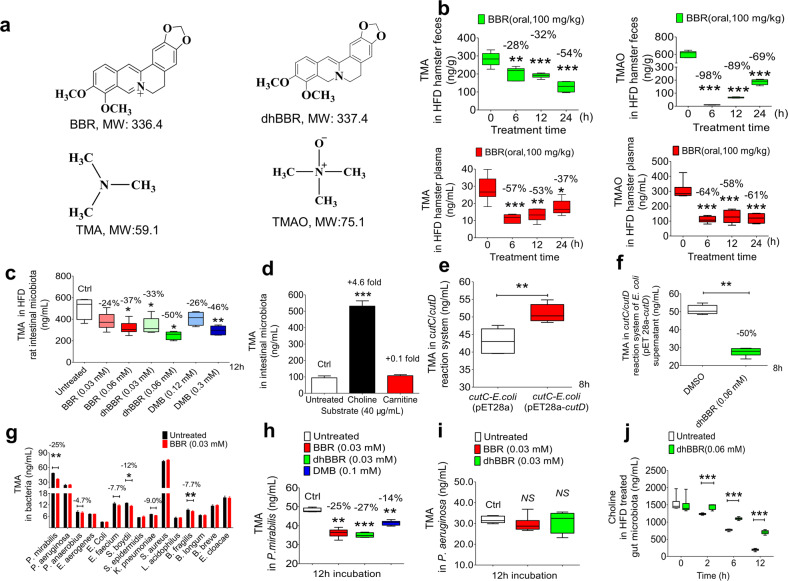
BBR decreased the production of TMA/TMAO in gut microbiota. **a** Chemical structures of berberine (BBR), dihydroberberine (dhBBR), trimethylamine (TMA) and trimethylamine-*N*-oxide (TMAO). **b** The levels of TMA and TMAO in faeces or plasma of the HFD-fed hamsters were significantly reduced 6, 12, and 24 h after oral administration of BBR (100 mg/kg, *n* = 6, *P < 0.05, **P < 0.01 and ***P < 0.001). **c** BBR, dhBBR (0.03, 0.06 mM) inhibited the production of TMA in the intestinal bacteria from HFD-fed SD rat, and the inhibitory effect of dhBBR was stronger than that of 3,3-dimethyl-1-butanol (DMB, 0.12, 0.3 mM), a positive control, after 12 h incubation (*n* = 6, *P < 0.05, **P < 0.01, ***P < 0.001). **d** A 4.6-fold increase of TMA was observed after addition of choline; but the phenomena was not seen with carnitine (40 μg/mL, n = 5, ***P < 0.001). **e** TMA level in the *cutC*-*E.coli* (transformed with pET28a-*cutD*) was elevated as compared to that in the *cutC*-*E.coli* (transformed with pET28a) (**P < 0.01) after 8 h incubation (**P < 0.01) (*n* = 6). **f** dhBBR (0.06 mM) inhibited the transformation from choline to TMA in a reaction system heterologously expressed choline-TMA lyase (*cutC*) (8 h, *n* = 6, **P < 0.01). **g** The effect of BBR (0.03 mM) on TMA production in 15 intestinal bacterial strains in vitro, and of the 15 strains, *P. mirabilis*, *S. boydii* and *B. fragilis* showed a significant decrease in TMA level after BBR treatment (*n* = 5, *P < 0.05, **P < 0.01). **h** BBR, dhBBR (0.03 mM) and DMB (a competitive inhibitor of choline-TMA lyase, 0.1 mM) decreased the level of TMA in *P. mirabilis* after 12 h incubation (*n* = 6, **P < 0.01, ***P < 0.001). **i** TMA level in *P. aeruginosa* did not change after BBR/dhBBR treatment (*n* = 6, 0.03 mM; *NS*, not significant). **j** DhBBR (0.06 mM) inhibited the decomposition of choline in the gut microbiota from the HFD fed rat (*n* = 6, ***P < 0.001). Data are expressed as mean ± SD and analysed with two-tailed student’s *t* test

Berberine (BBR) is an active compound isolated from Chinese traditional medicine *Coptidis Rhizoma* (Fig. 1a),^25^ and has been used to treat bacterial-caused diarrhoea as an over-the-counter (OTC) drug in China for decades. Since 2004, our group, as well as others, have identified BBR to be a safe and effective medicine for hyperlipidaemia and type 2 diabetes in clinic,^26–28^ with mechanisms and mode of action very different from the known drugs.^26,29^ The reported mechanisms include the up-regulation of low-density lipoprotein receptor (LDLR)^26^ and insulin receptor gene expression, activation of AMPK, inhibition of proprotein convertase subtilisin/kexin type 9 expression,^30^ and others.^31^ Although recent studies in animals have shown that BBR also exhibited inhibitory effect on plaque development in AS,^32,33^ little is known about the mechanism. The oral bioavailability of BBR is probably about 1%,^34,35^ suggesting the possible role of the gut microbiota in BBR’s therapeutic effect against AS.^9,36–41^

In what presented below we show that BBR could intrude the conventional operation of the choline-TMA-TMAO axis in the gut microbiota through inhibiting the activities of bacterial enzyme or coenzyme in this axis, and thus reduce the amount of TMA or TMAO in the intestine and circulation. Clinical study in AS patients provided supportive data, suggesting BBR a potential drug to decrease plaque development in AS.

## Results

### TMA/TMAO reduction in vivo by BBR was associated with the gut microbiota

We first established the targeted LC-MS/MS method for the quantification of both TMA and TMAO in biological samples (Supplementary Fig. S1a),^42^ and it showed that levels of TMA and TMAO could be accurately quantified by this method (Supplementary Tables S1–S7). The structures of TMA and TMAO are shown in Fig. 1a. After a single dose oral administration of BBR (100 mg/kg), TMA and TMAO levels in the HFD-fed hamsters significantly decreased in faecal samples, with 28 and 98% reductions at 6 h, 32 and 89% at 12 h, and 54 and 69% at 24 h, respectively (Fig. 1b; **P < 0.01, ***P < 0.001. Similar results were observed in plasma, in which the TMA level decreased by 57% at 6 h, 53% at 12 h, and 37% at 24 h; and for TMAO, the reductions were 64% at 6 h, 58% at 12 h, and 61% at 24 h after BBR treatment (Fig. 1b, *P < 0.05, **P < 0.01, ***P < 0.001)). While, there was not significant change observed after saline treatment at the same measurement point in 24 h (Supplementary Fig. S1b). To learn the role of the gut microbiota in reducing TMA/TMAO by BBR, BBR was administrated to the hamster intraperitoneously (*ip*, 20 mg/kg).^43^ Although the blood BBR in *ip* injection was higher than that in oral treatment (Supplementary Fig. S1c), the 24 h production of TMA and TMAO in faeces (Supplementary Fig. S1d) and plasma remained unchanged after BBR *ip* injection (Supplementary Fig. S1e). This result proved that TMA/TMAO reduction in vivo by BBR was associated with the gut microbiota. To confirm this hypothesis, the experiment of faecal transplantation (FT) was performed in HFD-fed hamsters. The bacteria isolated from HFD-fed or HFD-BBR-fed hamster faeces were fed to HFD-fed hamsters, respectively (Supplementary Fig. S2a). Results showed that after faecal transplantation of the gut microbiota treated with BBR (FT-HFD + BBR), the levels of TMA and TMAO in plasma and faecal samples were significantly reduced by comparison with the faecal transplantation group of HFD treated (FT-HFD), as shown in Supplementary Fig. S2b–e.

The above-mentioned results in vivo were supported by that in vitro. After incubating BBR with the gut microbiota from the HFD-fed rats for 12 h, TMA levels declined substantially in a dose-dependent manner, with 24% reduction at 0.03 mM and 37% at 0.06 mM (Supplementary Fig. S3a, *P < 0.05); the TMAO decrease was also significant, with 27% reduction at 0.03 mM and 37% at 0.06 mM (Supplementary Fig. S3a, *P < 0.05). TMA and TMAO were stable in the culture medium (Supplementary Fig. S4a, b). The results suggest that BBR might inhibit both TMA generation and the conversion from TMA to TMAO. Since a large percentage of oral BBR was transformed into dihydroberberine (dhBBR, Fig. 1a) by nitroreductase (NR) of the gut microbiota in the intestine,^37^ we also examined the effect of dhBBR on TMA and TMAO, in comparison with BBR. After incubating dhBBR with the gut microbiota from HFD-fed rat, both TMA and TMAO decreased dramatically at 12 h, with −33% and −50% decrease in TMA level and −49% and −59% decrease in TMAO when dhBBR concentration was at 0.03 and 0.06 mM, respectively (Supplementary Fig. S3b). BBR and dhBBR was stable in the bacterial culture medium under anaerobic condition for 8 h (Supplementary Fig. S4c, d). It appeared that the reduction of TMA and TMAO by dhBBR was more effective than that by BBR.

As intestinal TMA is produced mainly by bacterial choline-TMA lyase (CutC), CutC was investigated, with 3, 3-dimethyl-1-butanol (DMB), a non-lethal competitive inhibitor as reference. In the gut microbiota from rats, BBR or dhBBR (0.03 or 0.06 mM) decreased TMA production, as shown in Supplementary Fig. S3a, b; additionally, the CutC inhibitor DMB at high concentration inhibited TMA production in a potency (0.3 mM, −46%) comparable to that of low dose dhBBR (0.06 mM, −50%, Fig. 1c), suggesting dhBBR a strong CutC inhibitor. The result also identified CutC a possible target of dhBBR.

### DhBBR blocked TMA generation by inhibiting the bacterial CutC

In the intestine, TMA could generate by degrading choline or carnitine from food.^7,15^ CutC, as well as carnitine coenzyme A transferase (CCAT) is reported to be the key enzymes that catalyse TMA production, and CutC is activated in the presence of CutD, an activase of glycyl radical enzymes.^44^ In our experiment system, choline appears to be the main material for TMA production, because adding choline (40 μg/mL) into the alive bacterial culture elevated TMA level significantly (Fig. 1d); and this phenomena was not seen when carnitine was added (Fig. 1d). Addition of choline into the inactivated intestinal bacteria did not cause increase of TMA (Supplementary Fig. S5a). In addition, *E. coli* BL21 bacterial cell with *cutC* gene transformed with pet28a-*cutD* (Fig. 1e, Supplementary Fig. S7a) showed a promoted capacity to produce TMA as compared to the original *E. coli* transformed with pET28a. Moreover, dhBBR showed a substantial inhibition of TMA production in the *E. coli* with *cutC/cutD* genes (Fig. 1f), suggesting bacterial choline-TMA lyase (CutC) a target of dhBBR.

Then, BBR was incubated separately with 15 strains of intestinal bacteria, followed by TMA detection. In 3 out of 15 strains, *Proteus mirabilis* (*P. mirabilis*), *Shigella boydii* (*S. boydii*) and *Bacteroides fragilis* (*B. fragilis*), TMA decreased after treating with BBR for 12 h (0.03 mM, *P < 0.05, **P < 0.01, Fig. 1g), with the corresponding bacterial survival rate unchanged (Supplementary Fig. S5b), suggesting a co-existence of *cutC/cutD* gene in these three bacteria. Among the three strains, *P. mirabilis* expresses the *cutD* gene and the possible functional protein WP_004249185.1 of CutD.^16^ TMA was at a high level in the *P. mirabilis* culture, and was significantly supressed by BBR (−25%) or dhBBR (−27%) at their non-toxic concentration (0.03 mM, **P < 0.01, and ***P < 0.001; Fig. 1h, and Supplementary Fig. S5c). DMB was used as a positive control and decreased TMA by 14% (0.1 mM, **P < 0.01, Fig. 1h). In the *P. aeruginosa* strain, that has the functionally annotated gene of *CCAT*, TMA level remained unchanged after BBR/dhBBR (0.03 mM) treatment (Fig. 1i, Supplementary Fig. S5d), suggesting BBR/dhBBR a regulator for CutC, but not CCAT, in TMA biosynthesis (also see Fig. 1d).

**Fig. 2:**
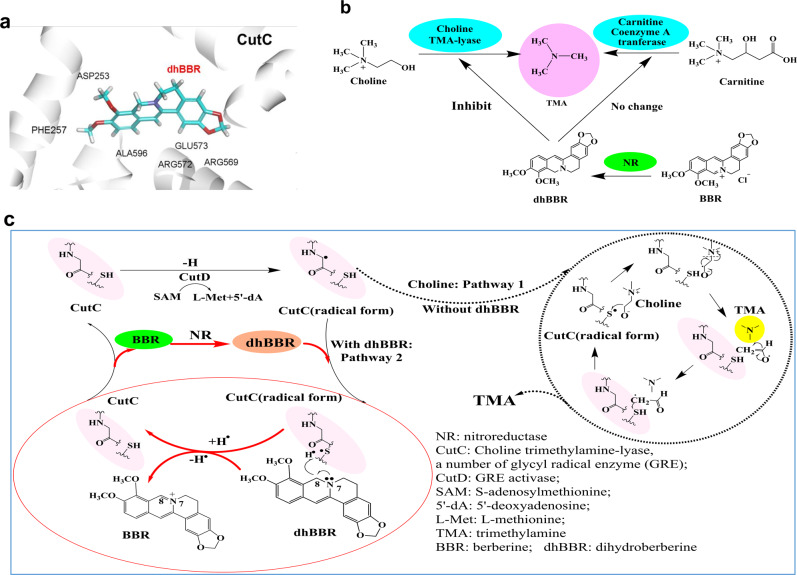
Putative mechanisms of dhBBR on inhibiting bacterial choline-TMA lyase (CutC). **a** The 3D docking results of dhBBR and bacterial CutC by CDOCKER tool showed that multiple interactions might mediate the binding of dhBBR into the pocket. **b** BBR inhibited TMA production through its characteristic metabolite dhBBR from gut microbiota, and the inhibition was on CutC rather than CCAT. **c** Assumed mechanism of dhBBR in down-regulating CutC

The putative molecular details are shown in Fig. 2. First, virtual docking between dhBBR (or BBR) and the enzyme CutC (or CCAT) was done with the CDOCKER. The result showed that dhBBR was likely to bind the active site of CutC, via multiple interactions of the carbon hydrogen bond, Van der Waals, Pi-alkyl and alkyl interaction et al. with the CDOCKER energy of −22.10 kJ/mol (Fig. 2a, and Supplementary Fig. S6a, b), while the docking energies between BBR and CutC (or CCAT) were rather high, with CDOCKER energies of 38.12 and 18.43 kJ/mol, respectively, in Supplementary Fig. S6a). The predicted interaction between dhBBR and amino acids in the binding pocket is shown in Supplementary Fig. S6b. It suggested a weak interaction between BBR and CutC. A feeble interaction was also seen between dhBBR and CCAT (in Supplementary Fig. S6a). The predicted action mode of dhBBR on the CutC is demonstrated in Fig. 2b and c. Biochemically, the glycyl radical activating enzyme CutD (activase of CutC) can generate a glycyl radical on CutC, and the radical of the active CutC locates on the alpha site of the carbonyl group.^44^ In the presence of choline (Fig. 2c, pathway 1), the radical of alpha site of the carbonyl group could obtain the active hydrogen from the sulfhydryl group and make the S atom possess a radical in CutC.^44,45^ Then, C atom on the hydroxy ortho-methylene of choline is susceptible to provide an H^•^ to the S^•^ of sulfhydryl group of CutC, which causes the choline an electron rearrangement and breaks off from the bond of C-N and produce TMA (Fig. 2c); and CutC (radical form) acquires an H^•^ and returns to its stable form (CutC).^44^ However, in the presence of dhBBR (Fig. 2c), since the binding affinity of dhBBR to CutC was strong (in Supplementary Fig. S6a), CutC (radical form) prefers to get an H^•^ from 8-C of dhBBR and returned to the stable CutC (Fig. 2c, pathway 2). Thus, dhBBR inhibited choline fragmentation by consuming more active CutC (radical form) and thus blocks the formation of TMA (Fig. 2c). At the same time, dhBBR lost an H^•^ and was oxidized back to BBR (Fig. 2c). Indeed, bioassay showed that choline was largely stabilized by dhBBR in our experimental system, suggesting a good interaction between CutC and dhBBR, stronger than that between choline and CutC (Fig. 1j).

### DhBBR inhibited TMAO generation by targeting flavin-containing monooxygenases (FMOs) in the gut bacteria

FMO_3_ is the key enzyme that participates in the production of TMAO in liver.^19^ As TMAO was detectable in faeces (Figs. 1b and 4a), we assumed that FMOs-like enzymes might express in the gut bacteria and contribute to the biosynthesis of TMAO from TMA in the intestine. As shown in Fig. 4a, the production of TMAO in vitro was significantly inhibited by BBR (−34%, 0.03 mM; −47%, 0.06 mM), dhBBR (−49%, 0.03 mM; −72%, 0.06 mM) and the FMO inhibitor imipramine (−58%, 0.03 mM; −84%, 0.06 mM) in the gut micobiota (***P < 0.001), indicating the presence of FMOs in the intestinal bacteria. TMAO level was also decreased by methimazole, another inhibitor of bacterial FMO, but with less activity as compared to imipramine (−29%, *P < 0.05, 0.1 mM; −44%, ***P < 0.001, 0.33 mM; Fig. 4a). Then, fifteen intestinal bacterial strains were treated separately with BBR (0.03 mM). TMAO was detected in 4 of the 15 strains, which (*P. mirabilis*, *Pseudomonas aeruginosa, Peptostreptococcus anaerobius, and Enterobacter aerogenes*; Fig. 4b, and Supplementary Fig. S5b) are positive for the *fmo* gene (NC_002516.2: c1677445-1675862, and the predicted corresponding protein: NP_250229.1, Supplementary Fig. S7b).^46^ In *P. aeruginosa*, after treating with BBR or dhBBR (0.03 mM), the generation of TMAO was reduced by −27% and −31%, respectively (*P < 0.05, **P < 0.01, Fig. 4c), without inhibition of the bacterial growth (Supplementary Fig. S5d). Imipramine (0.1 mM) showed a 37% decrease of TMAO (**P < 0.01, Fig. 4c). Then, the *fmo* gene of *P. aeruginosa* was transformed into an *E. coli* strain (BL21, pET28a-*fmo*) (Supplementary Fig. S7b, c), and the corresponding protein in the supernatant showed the expected oxidation function of making TMAO (Fig. 4d). After incubation in the FMO-containing reaction system, the conversion from TMA to TMAO was inhibited by 67% after dhBBR treatment (0.06 mM, *P < 0.05, Fig. 4e), confirming the good inhibitory effect of dhBBR on the activity of bacterial FMO. To learn BBR’s or dhBBR’s direct effect on FMO, liver homogenate was used as it contains FMO but not CutC. After incubating dhBBR or BBR with the liver homogenate, TMAO production significantly decreased by 40% with dhBBR or 16% with BBR in 2 h at the low-dose, and 57% (dhBBR) or 37% (BBR) in the high-dose (**P < 0.01; ***P < 0.001, Supplementary Fig. S5e), similar to that of imipramine. The results demonstrated dhBBR (or BBR) a good inhibitor for TMAO production working via direct suppression of FMOs activity.

**Fig. 3:**
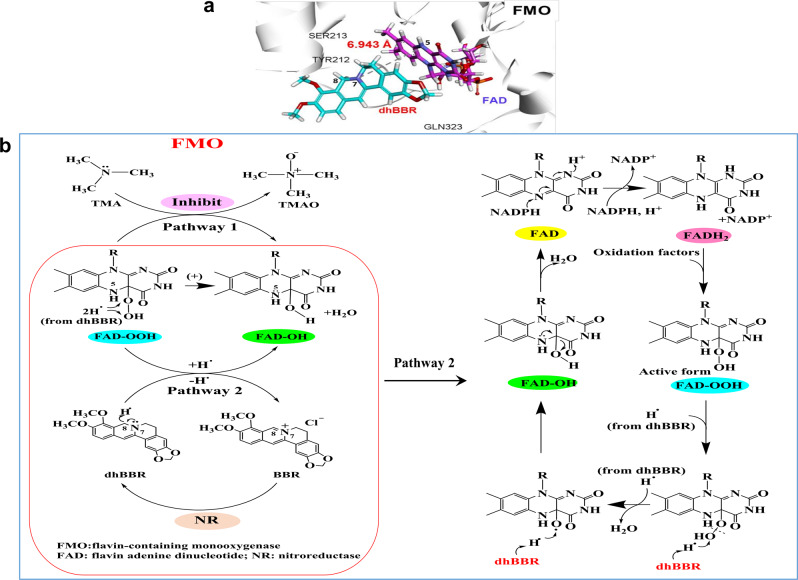
Putative mechanisms of dhBBR on inhibiting bacterial flavin-containing monooxygenase (FMO). **a** The virtual docking of dhBBR with bacterial FMO by CDOCKER tools and the possible interactions. **b** BBR might inhibit TMAO production through dhBBR’s inhibitory activity on FMO in the gut bacteria

Then, the biochemical mechanisms of dhBBR (and BBR) in inhibiting FMOs were explored using CDOCKER virtual docking analysis. As compared with BBR, dhBBR exhibited a better docking performance onto bacterial FMO with a binding free energy of −41.8 kJ/mol, lower than that by TMA (−9.45 kJ/mol), suggesting a high affinity between dhBBR and FMO. Additionally, dhBBR could anchor into the binding site of FMO through multiple interactions with its scaffold and side chains, including carbon hydrogen bond, conventional hydrogen bond, Van der Waals, Pi-alkyl, and alkyl interactions, et al. The amino acids possibly interacts with dhBBR is shown in Fig. 3a and Supplementary Fig. S6c. The detailed binding analysis is shown in Fig. 3b. In the active catalysing pocket, the distance between the 7-N atom of the C-N bond in dhBBR and the 5-N atom of the coenzyme flavin adenine dinucleotide (FAD) was 6.943 Å, less than 10 Å and suitable for electron transfer. In the conversion from TMA to TMAO, the oxygenated form of coenzyme FAD (4α-hydroperoxyflavin, FAD-OOH in Fig. 3b) plays a key role,^47^ and the binding pocket in FMO for dhBBR is also the one for FAD. Therefore, the molecular mechanism of dhBBR on inhibiting FMO is predicted as following. As dhBBR is more capable of providing H^•^ than TMA does, when dhBBR approaches the coenzyme in the FMO pocket, the O atom of -OH in the peroxy bond of FAD-OOH is more likely to obtain H^•^ from the 8-C of dhBBR, which caused the breakdown of the peroxy bond and resulted in the production of H_2_O and FAD-OH (4α-hydroxyflavin, in Fig. 3b, pathway 2). Due to the instability of FAD-OH, it is easy to obtain H^•^ from dhBBR and remove a H_2_O; accordingly, FAD-OH is converted into FAD (Fig. 3b). Therefore, some FAD-OOH can react with dhBBR, instead of TMA (Fig. 3b, pathway 1), thus, the formation of TMAO is restricted. Meanwhile, dhBBR loses an H^•^ and oxidized into BBR (Fig. 3b).

**Fig. 4:**
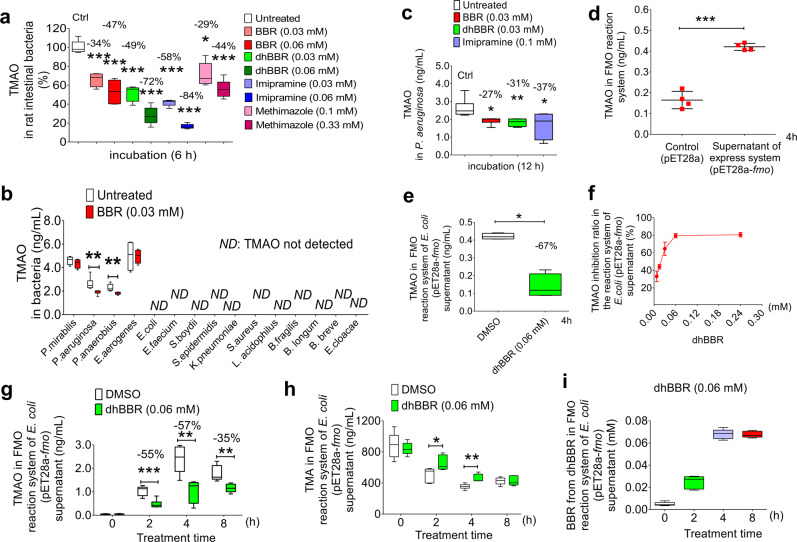
DhBBR inhibited TMAO generation by targeting bacterial flavin-containing monooxygenase (FMO) in gut. **a** Both BBR and dhBBR (0.03, 0.06 mM) inhibited TMAO production in intestinal bacteria (*n* = 5, ***P < 0.001), and the known FMO inhibitors (imipramine and methimazole) showed an inhibition as well. **b** The effect of BBR (0.03 mM) on TMAO production in vitro was tested in 15 intestinal bacterial strains, of which TMAO was detected in *P. mirabilis*, *P. aeruginosa*, *P. anaerobius* and *E. aerogenes*. In *P. aeruginosa* and *P. anaerobius*, TMAO showed a decrease after BBR treatment (*n* = 5, **P < 0.01). **c** BBR, dhBBR (0.03 mM) and imipramine (inhibitor of FMO, 0.1 mM) decreased TMAO level in *P. aeruginosa* after incubation for 12 h (*n* = 6, *P < 0.05, **P < 0.01). **d** The conversion from TMA to TMAO increased in the FMO-expressed reaction system (pET28a-*fmo*) after 4 h incubation (*n* = 4, ***P < 0.001). **e** BBR and dhBBR (0.06 mM) inhibited the transformation from TMA to TMAO in a heterologously FMO-expressed reaction system (*E. coli* with pET28a-*fmo* transformation) at 4 h after incubation (*n* = 4, *P < 0.05). **f** The inhibition ration (%) of TMAO production in the FMO-reaction system (in *E. coli* with pET28a-*fmo*) was dose-dependent (*n* = 4). **g**, **h** DhBBR (0.06 mM) inhibited the production of TMAO (**g**) and increased the level of TMA (**h**) in the FMO-expression reaction system (*E. coli* pET28a-*fmo*, *n* = 6, *P < 0.05, **P < 0.01, ***P < 0.001). **i** DhBBR was transformed into BBR in the FMO reaction system (*E. coli* pET28a-*fmo*), *n* = 6. Data shown are mean ± SD and analysed with two-tailed student’s *t* test

To confirm the mechanisms, an in vitro bacterial FMO reaction system was employed in the study. As shown in Fig. 4f, g, the inhibitory effect on TMAO by dhBBR was in a time- and dose-dependent manner; accordingly, TMA level was stabilized by dhBBR (Fig. 4h). At the same time, dhBBR transformed to BBR (Fig. 4i). The results suggested dhBBR an inhibitor for TMAO production, working through control of FMOs in the gut microbiota. The elucidation of crystal structure of dhBBR binding to enzyme/co-enzymes has not been completed yet, due to the poor water solubility of BBR/dhBBR. Active research is going on in our laboratory.

### BBR ameliorated AS in animal models through inhibiting TMAO production in the gut microbiota

Hamsters were used because their lipoprotein profile and aortic lesion morphology are similar to that in humans.^48^ Animal model of AS was established by feeding hamsters with HFD for 10 months.

**Fig. 5:**
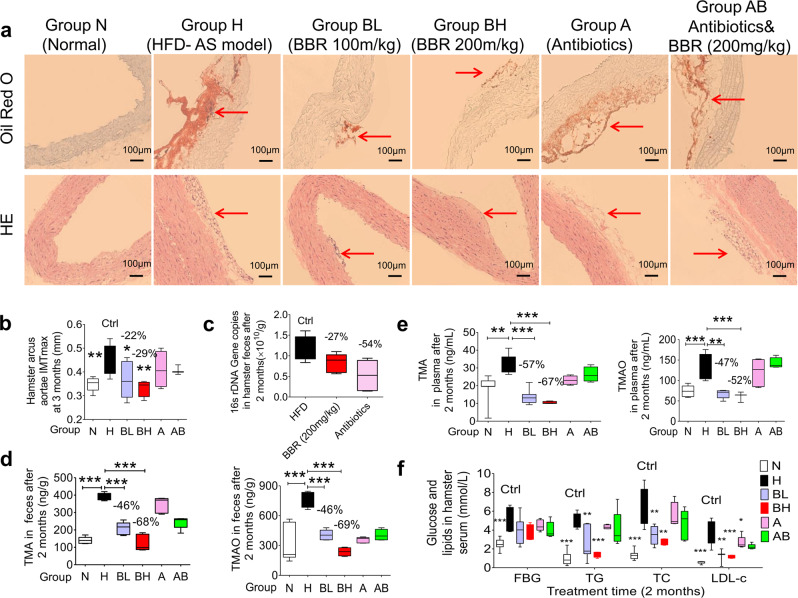
Anti-atherosclerotic effects of BBR in hamsters fed with high-fat diet. **a** Oil red O and HE staining of arcus aortae in atherosclerotic hamsters after BBR treatment for 3 months. Group N, the normal control group, *n* = 8; Group H, the atherosclerosis model group fed with HFD, *n* = 7; Group BL, the low-dosage BBR group (oral, 100 mg/kg/d), *n* = 7; Group BH, the high-dosage BBR group (oral, 200 mg/kg/d), n = 4; Group A, the group treated with antibiotics (oral, terramycin 300 mg/kg/d, erythromycin 300 mg/kg/d and cefadroxil 100 mg/kg/d, 3 months), n = 5; Group AB, the group treated with BBR and the combination of antibiotics (oral, terramycin 300 mg/kg/d, erythromycin 300 mg/kg/d, cefadroxil 100 mg/kg/d, and BBR 200 mg/kg/d; 3 months), *n* = 5. Red arrows: the location of plaques. **b** The maximum intima-media thickness (IMT_max_) measurement of arcus aortae in hamsters showed that BBR significantly alleviated the severity of atherosclerosis after 3 months treatment (*P < 0.05, **P < 0.01). **c** 16 s rDNA Gene copies in hamster faeces at the point of two months. The number of colonies was decreased after the treatment with combination of antibiotics for two months (−54%) or the treatment with BBR (−27%, 200 mg/kg), in comparison with the HFD model group. **d** BBR significantly inhibited the production of TMA and TMAO in faeces, and the effects were weakened after intervention with antibiotics (2 months, ***P < 0.001). **e** Oral administration of BBR significantly lowered the levels of TMA and TMAO in plasma after 2 months therapy (**P < 0.01, ***P < 0.001)**. f** The levels of FBG, TG, TC and LDL-C in serum were significantly lowered by oral administration of BBR for 2 months (*P < 0.05, **P < 0.01, ***P < 0.001). Data in **b**–**f** are expressed as the mean ± SD and analysed with two-tailed student’s *t* test

37 hamsters were divided into six groups, namely: Group N, the normal control group; Group H, the HFD-induced AS model group; Group BL, the AS model hamsters treated with low dose BBR (oral, 100 mg/kg/d); Group BH, the AS model hamsters treated with high dose BBR (oral, 200 mg/kg/d); Group A, the AS model hamsters orally treated with mixed antibiotics (terramycin 300 mg/kg/d, erythromycin 300 mg/kg/d and cefadroxil 100 mg/kg/d); Group AB, the AS model hamsters treated with both antibiotics and BBR (oral, 200 mg/kg/d). After treating AS hamsters with BBR for 3 months, all of the animals were sacrificed and aortic vessels were collected for histological staining. Oil red O and hematein & eosin (HE) staining were used to view the atherosclerotic plaques, in which the lipid rich plaques were stained red with oil red O and showed vacuoles in HE staining. The histological results showed that plaques were obviously stained with oil red O in Group H (Fig. 5a), while the vessels in Group N were smooth inside the cavity of aortic arch and free of oil red O staining (Fig. 5a). In the HFD-fed hamsters treated with BBR for 3 months (100 mg/kg/d or 200 mg/kg/d), staining signal in the atherosclerotic plaques was significantly less than that in Group H (Fig. 5a). In contrast, the anti-plaque efficacy in animals treated with antibiotics (Group A) or antibiotics plus BBR (Group AB) was much less (Fig. 5a). In the anti-plaque evaluation, the maximum intima media thickness (IMT_max_) of the aortic arch is also a crucial indication,^49^ and increase of which indicates an aggravation of vessel lesion in AS. The IMT_max_ index in hamsters of Group H was significantly higher than that in Group N (**P < 0.01, Fig. 5b), indicating a success of the AS model. The IMT_max_ value of hamster aortic arch was largely reduced by BBR after 3-month on treatment, by 22% in the Group BL (*P < 0.05) and 29% in the Group BH (**P < 0.01, Fig. 5b), verifying the anti-plaque effect of BBR for AS. However, in the groups treated with antibiotics or antibiotics plus BBR, the IMT_max_ value did not decline significantly (Group A and Group AB, Fig. 5b), because treatment with antibiotics for 3-month inhibited the bacterial number in the gut flora (−54%, Fig. 5c), further demonstrating the importance of gut microbiota. At the same time, BBR decreased the bacterial colonies by −27% at the dose of 200 mg/kg (Fig. 5c). The results showed that the BBR is a drug by regulating bacteria but not a completely antibiotics.

The consumption of choline in gut microbiota increased in the atherosclerosis model group (Group H) compared with the normal control group (Group N, Supplementary Fig. S8a), and the corresponding TMA and TMAO levels significantly increased in the feces and plasma (Fig. 5d, e). After 2 months of BBR treatment, the left choline in gut microbiota significantly increased and thereby TMA and TMAO levels in faeces were remarkably reduced by −46% and −46% in the Group BL (***P < 0.001, in Supplementary Fig. S8a and Fig. 5d), and −68% and −69% in the Group BH (***P < 0.001, in Fig. 5d). Accordingly, a decrease of TMA and TMAO in plasma was evidenced in parallel, with −57% and −47% in the Group BL and −67% and −52% in the Group BH, respectively (**P < 0.01, ***P < 0.001, Fig. 5e). Plasma choline level in vivo decreased after BBR treatment (Supplementary Fig. S8b). However, moderate reduction of TMA and TMAO was observed in the Group AB (Fig. 5d, e). In addition, blood glucose and lipid levels declined after BBR treatment, with *P* values less than 0.01 or 0.001, confirming the systemic effect of BBR (Fig. 5f). While, in the antibiotics treated group (AB), the regulating capacity of BBR in the glucose and lipids profiles was weak, which might be attribute to that the intestinal bacteria play important roles in the absorption of BBR. Antibiotics treatment could reduce the bioavailability of BBR.^37^ The reduction in BBR level at the in vivo target might correspondingly weaken the efficacy of BBR in lowering blood glucose and lipids. By principle, this systemic effect of BBR favours anti-AS treatment. In addition, the results of FMO level in liver showed that the enzyme was up-regulated in the atherosclerosis hamster model, and BBR or antibiotics did not significantly influence the FMO level in liver. The result suggested that BBR reduced the TMA and TMAO level was mainly due to the intestinal bacteria (Supplementary Fig. S8c).

Composition analysis for the intestinal bacteria verified the regulatory effect of BBR on gut microbiota (Supplementary Fig. S8d, e). Oral BBR adjusted the abundance of bacterial species nearly to the normal level (Supplementary Fig. S8d). Among the top 50 genera that changed the most (Supplementary Fig. S8e), 13 showed a significant decrease after oral BBR, namely, *Eubacterium_coprostanoligenes*, *Treponema_2*, *Ruminococcaceae_UCG-002*, *Prevotellaceae_UDG-001*, *Flavobacterium*, *Candidatus_Saccharimonas, Empedobacter*, *Corynebacterium_1*, *Jeotgalicoccus*, *Myroides*, *Kurthia*, *Acinetobacter and Ruminoccoccus_2* (marked in blue “*” in Supplementary Fig. S8e). Among them, *Acinetobacter*, *Kurthia*, *Prevotellaceae_UDG-001*, and *Ruminococcaceae_UCG-002*, the known TMA producer,^50^ decreased in abundance after BBR treatment (Supplementary Fig. S8f-i); reduction of these bacteria by BBR might contribute to the reduced level of TMA in gut microbiota. Additionally, *Eubacterium*, a genus that has been reported to generate TMA with CutC,^51^ was also decreased. Moreover, the probiotics *Allobaculum*, *Akkermansia* and *Lachnospiraceae_NK4A136*, which are known producers of short-chain fatty acids,^39,52,53^ increased in abundance (marked in red “*” in Supplementary Fig. S8e), and they might cause harmony effect on the metabolism of glucose and lipids as well.^54^ Thus, remodelling the structure of gut microbiota could also be a component of BBR in its action of reducing TMA and TMAO.

### BBR alleviated atherosclerosis in patients with AS

As BBR is an OTC drug in China, it was then translated into clinical application. 49 patients were enrolled in the BBR treatment study in the Outpatient Section of the First Hospital of the Jilin University in Changchun in early spring of 2017 (Clinical approval number ChiCTR-OPN-17012942). 16 of them (age 60.5 ± 8.9; 7 males and 9 females) had their blood lipids and glucose in the normal range, with total cholesterol (TC, mmol/L) of 4.59 ± 0.57, triglyceride (TG, mmol/L) of 1.12 ± 0.37, low density lipoprotein-c (LDL-C, mmol/L) of 2.49 ± 0.41 and fasting blood glucose (FBG, mmol/L) of 5.16 ± 0.49; these individuals were in Group 1 and served as reference of TMA and TMAO levels with respect to that of the AS patients described below (Supplementary Table S7). 21 individuals were hyperlipidaemia patients (age 63.7 ± 5.2; 12 males and 9 females) with high levels of blood glucose or lipids [TC (mmol/L), 5.70 ± 1.04; TG (mmol/L), 3.65 ± 4.55; LDL-C (mmol/L), 3.11 ± 0.85; FBG (mmol/L), 6.79 ± 2.29] as Group 2 (Supplementary Table S7). The 21 hyperlipidaemia patients were diagnosed with AS and not undergoing any hypolipidemic treatment before enrolling the study. BBR was given orally to the 21 patients for 4 months (1 gram per day). In parallel, other 12 AS patients were enrolled as reference of known drugs [Group 3, age 55.60 ± 8.85; 10 males and 2 females]. Their baseline was as following, TC (mmol/L), 4.53 ± 1.10; TG (mmol/L), 1.54 ± 0.51; LDL-C (mmol/L), 2.77 ± 0.73; and FBG (mmol/L), 7.71 ± 3.44; they were treated with rosuvastatin plus aspirin, with clopidogrel sulfate or ticagrelor in the regimen if necessary, according to the guidelines for treating CVD.^55^ The baseline characteristics of the clinical participants are shown in Supplementary Table S8.

**Fig. 6:**
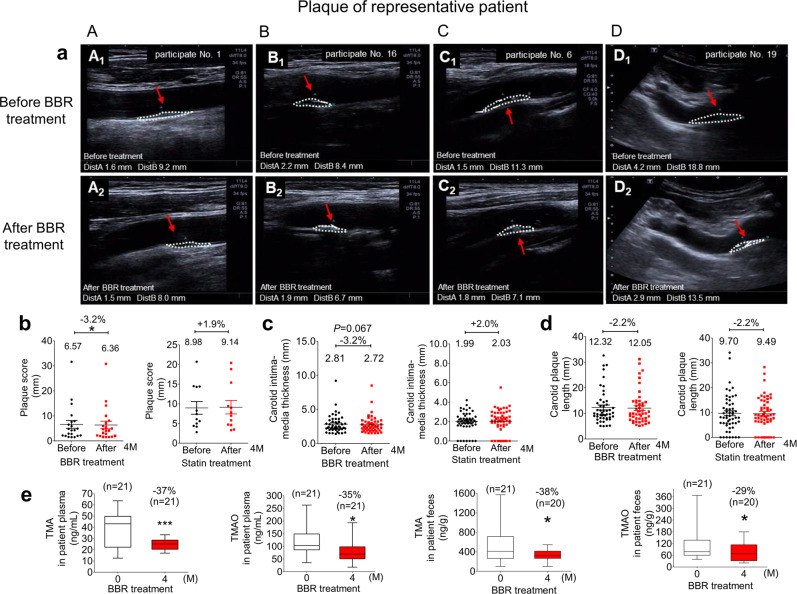
BBR reduced the plaque size in patients with atherosclerosis. **a** The ultrasonic images of atherosclerotic plaques (circle in white) of the patients at different positions of carotid arteries before (1) and after (2) 4-month BBR therapy are shown, including the common carotid artery (A: participate No. 1, plaque No. 1), the carotid bifurcation (B: participate No. 16, plaque No. 38), the internal carotid artery (C: participate No. 6, plaque No. 19) and the subclavian artery (D: participate No. 19, plaque No. 44). Also, please see Table S9 in the supplemental material. **b** The average plaque score in patients was decreased by 3.2% after oral BBR (0.5 g, bid) for 4 months (*P < 0.05, *n* = 21); the score value was slightly increased (by +1.9%) after treatment with conventional drug combination, including rosuvastatin, aspirin, as well as clopidogrel sulfate or ticagrelor (*n* = 12) if needed. **c** The average carotid intima-media thickness was reduced by 3.2% after oral treatment with BBR (*P* = 0.067), and the value was slightly increased (by +2.0%) in the combination therapy. **d** The average carotid plaque length in patients was decreased by 2.2% in either oral BBR (0.5 g/bid) group or combination drug group after 4 months on therapy. **e** TMA and TMAO level in faeces and plasma samples of patients went down after 4 months BBR treatment; the TMA/TMAO levels in plasma were lowered by 37% / 35% (***P < 0.001; *P < 0.05), and TMA/TMAO levels in faeces decreased by 38%/29% (*P < 0.05; *P < 0.05). Data shown are the mean ± SEM and analysed with one-tailed paired *t* test

The faecal sample test showed 88% increase of TMA (282.8 ± 109.7 *vs* 533.2 ± 362.5 ng/mL, **P < 0.01) and 63% of TMAO (67.0 ± 38.0 *vs* 109.1 ± 79.7, ng/mL, *P < 0.05) in the Group 2 patients, as compared with Group 1 (Supplementary Fig. S9a). Accordingly, the plasma level of TMA and TMAO in the Group 2 were higher than that in Group 1, by 6% (37.0 ± 12.5 *vs* 39.4 ± 15.8 ng/mL, Supplementary Fig. S9b, *P* = 0.13) for TMA and 14% for TMAO (106.6 ± 52.0 *vs* 121.8 ± 57.5 ng/mL; Supplementary Fig. S9b, *P* = 0.31). With BBR treatment (oral, 1 gram a day, 4 months), blood glucose and lipids of the Group 2 patients decreased [TC (mmol/L): 5.70 ± 1.04 *vs*. 5.24 ± 0.83, **P < 0.01; TG (mmol/L): 3.65 ± 4.55 *vs*. 2.73 ± 4.00, *P < 0.05; LDL-C (mmol/L): 3.11 ± 0.85 *vs*. 2.99 ± 0.75; FBG (mmol/L): 6.79 ± 2.29 *vs*. 6.39 ± 2.39] (Supplementary Table S8). The average levels of TC and LDL-C returned to the normal range (Supplementary Table S7), confirming the systemic therapeutic efficacy of BBR for blood lipids and glucose. TMA in plasma was reduced by 37% (39.37 ± 15.78 *vs*. 24.76 ± 4.66, ng/mL, ***P < 0.001) and TMAO by 35% (121.84 ± 57.49 *vs*. 79.53 ± 43.57, ng/mL,*P < 0.05) after 4 months on BBR therapy (Supplementary Table S8, Fig. 6e). Significant TMA/TMAO decrease in patient faeces was seen as well (38%↓, 533.18 ± 362.49 *vs*. 332.38 ± 119.56 ng/mL for TMA level, *P < 0.05; 29%↓, 109.05 ± 79.67 *vs*. 77.10 ± 47.54 ng/mL for TMAO, *P < 0.05) (Supplementary Table S9, Fig. 6e). The composition of the gut microbiota in patients was restructured after oral BBR. The 16 S rRNA results revealed that 11 of the top 50 genera that changed the most showed a decrease in abundance (marked with a red “*”, Supplementary Fig. S10a). Among the 11 genera, *Eubacterium_hallii_group, Anaerostipes*, *Faecalibacterium, Dialister*, *Eubacterium_coprostanoligenes_group, Coprococcus_3, Butyricicoccus* and *Clostridium_sensu_strito_1* (marked with a red “Δ”, Supplementary Fig. S10a), had the potential to produce TMA.^50,51^ The abundance of these bacterial species were significantly declined by BBR (Supplementary Fig. S10b); for instance, *Eubacterium_coprostanoligenes_group*, was decreased by 63% (**P < 0.01), *Eubacteriu_hallii_group* by 69% (*P < 0.05), *Dialister* by 95% (**P < 0.01), *Clostridium_sersu_stricto_1* by 75% (**P < 0.01)*, Faecalibacterium* by 48% (*P < 0.05), *and Butyricicoccus* by 50% (**P < 0.01). The results agreed with that from animal experiment (Supplementary Fig. S8e).

Using ultrasonography, the therapeutic efficacy of BBR for plaques in AS patients was investigated by calculating the plaque scores of each patient before and after drug treatment at 12 positions of carotid artery,^56^ including the bilateral common carotid artery, bilateral carotid bifurcation, bilateral internal carotid artery, bilateral external carotid artery, bilateral vertebral artery intervertebral space and bilateral subclavian artery. Additionally, other methods including the carotid plaque length and the brachial-ankle pulse wave velocity (baPWV) were also used to access the severity of vascular lesions (Supplementary Fig. S9c, d).^49,57,58^ In this investigation, every plaque of each patient was examined and analysed before and after treatment in order to determine the therapeutic efficacy. Of the 21 AS patients treated with BBR, one patient had 8 plaques and was the most severe case in this group (with plaque score of 31.6 mm); one had 6 total plaques on both sides (plaque score 16.5 mm), one had 5 plaques (score 14.8 mm), and one had 4 plaques (score 10.2 mm). Other 2 subjects had 3 plaques (score 5.9 mm and 7.6 mm), 5 subjects had 2 plaques and 10 had one plaque (Table 1 and Supplementary Table S10). Thus, in the Group 2, total 49 plaques with different scales were investigated for the treatment efficacy (Supplementary Table S9). Representative image of the plaques (A, B, C, D) before and after BBR treatment are shown in Fig. 6a. By measuring the carotid intima-media thickness (mm), as well as the carotid plaque length (mm) of each plaque, 28 out of 49 plaques was reduced in size after BBR treatment, such as in image A, common carotid artery, 1.6 × 9.2 mm *vs* 1.5 × 8.0 mm; B, carotid bifurcation, 2.2 × 8.4 mm *vs* 1.9 × 6.7 mm; C, internal carotid artery, 1.5 × 11.3 mm *vs* 1.8 × 7.1 mm; and D, subclavian artery, 4.2 × 18.8 mm *vs* 2.9 × 13.5 mm (Fig. 6a, A1-A2; B1-B2; C1-C2; D1-D2). To quantify the severity of plaque, plaque scores (mm) were calculated by the sum of the thickness of the plaques per patient.^56^ The average plaque score of the 21 patients showed a significant decrease by 3.2% after BBR treatment, from 6.57 to 6.36 mm (*P < 0.05, Fig. 6b; Table 1), suggesting a potential anti-plaque effect of BBR in AS patients. The carotid intima-media thickness, as well as the average carotid plaque length reduced by 3.2% and 2.2%, respectively (Fig. 6c, d). Apart from this, bilateral baPWV showed a minor decline (−1.2% and −2.0%), suggesting a reduced development of plaques by BBR. By counting the patients, 12 out of the 21 patients (57% of the cohort) had their plaque score reduced, 3 showed no change and 6 showed increased plaque score (Table 1).

**Table 1:** Plaque scores of the atherosclerosis after drug treatment*

Drug	No.	Code	Plaque scores before	Plaque scores after	Δ	% of reduction
			treatment^a^ (mm)	treatment^b^ (mm)	(b-a)	(b-a)/a*100
BBR	1	Y GY	1.6	1.5	−0.1	–
	2	G DW	14.8	14.1	−0.7	–
	3	L JM	5.9	6	0.1	–
	4	L ZF	4.8	4.9	0.1	–
	5	C K	7.6	7.1	−0.5	–
	6	T WM	16.5	15.8	−0.7	–
	7	W YH	2.0	2.0	0	–
	8	L KJ	4.3	4.1	−0.2	–
	9	K LH	1.5	1.5	0	–
	10	Z LY	2.0	1.6	−0.4	–
	11	L CX	2.9	2.8	−0.1	–
	12	F XL	4.2	4.1	−0.1	–
	13	L R	1.5	1.7	0.2	–
	14	W SC	2.8	3.2	0.4	–
	15	C JR	31.6	30.8	−0.8	–
	16	C LJ	2.2	1.9	−0.3	–
	17	Y XY	7.9	8.1	0.2	–
	18	S HB	4.9	5.4	0.5	–
	19	Y CR	2.2	2.2	0	–
	20	S LT	10.2	10	−0.2	–
	21	Q SF	6.5	4.7	−1.8	–
	Mean		6.57	6.36	−0.21	−3.2
Statins	1	W JT	6.1	5.8	−0.3	–
	2	Z WT	14.3	9.4	−4.9	–
	3	H Z	14.4	18.8	4.4	–
	4	L SQ	5.0	4.7	−0.3	–
	5	Z YL	8.3	7.8	−0.5	–
	6	L SH	4.2	4.6	0.4	–
	7	L W	2.8	3.5	0.7	–
	8	S BJ	5.6	9.0	3.4	–
	9	L JL	7.4	7.0	−0.4	–
	10	L CL	14.5	20.4	5.9	–
	11	X ZQ	20.7	15.4	−5.3	–
	12	L ZE	4.4	3.3	−1.1	–
	Mean		8.98	9.14	0.17	1.9

^#^China Clinical Trial Registry: ChiCTR-OPN-17012942.

As positive drug reference, 12 patients were treated with statin plus aspirin (Supplementary Table S7), according to the European Society of Cardiology and World Heart Federation.^55^ Among these 12 patients, 2 had ten plaques on both sides of carotid artery with plaque scores of 20.7 mm, and 14.5 mm, respectively; 2 had six plaques (plaque scores 14.3 mm and 14.4 mm), 1 had five plaques (score 5.6 mm), and 1 had four plaques (score 8.3 mm). Others had 1–3 plaques (Table 1 and Supplementary Table S11). Thus, in total, 54 plaques were investigated in this group (Supplementary Table S11). The results showed that the guided therapy with statin and aspirin for 4 months might alleviate the development of plaques with a slight increase of 1.9% for the average of plaque score (from 8.98 to 9.14 mm) (Fig. 5b; Table 1). The carotid intima-media thickness, as well as the average carotid plaque length showed a 2.0% increase and a −2.2% decrease, respectively (Fig. 6c, d). Considering AS plaque a continuously progressive pathogenesis course, oral BBR might suppress plaque development in AS patients. Among the patients treated with the positive drug reference, 7 out of the 12 exhibited reduced plaque score (58% of the cohort), 0 showed no change and 5 had their plaque score increased (Table 1). The results were similar to those treated with BBR.

## Discussion

**Fig. 7:**
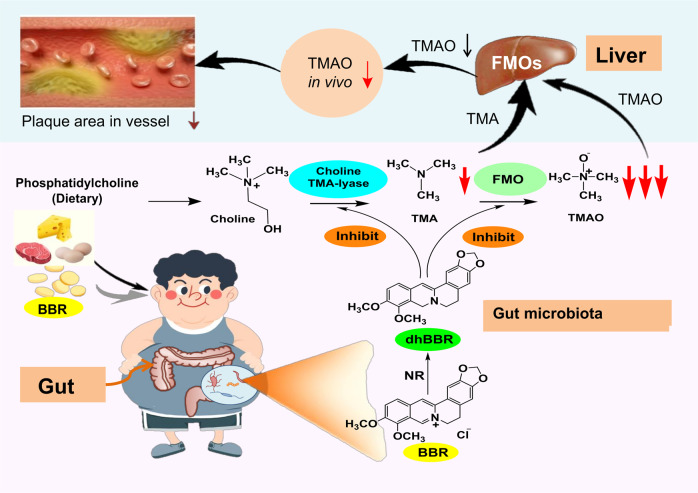
Berberine (BBR) treats atherosclerosis through its vitamin-like effect down-regulating TMAO production in the gut microbiota

In the present study, we show that BBR could decrease the levels of TMA and TMAO in faeces and blood, improved blood lipid profiles and significantly decreased IMT_max_ value in the HFD-fed arteriosclerosis hamsters. Further investigation revealed that BBR decreased the transformation either from choline to TMA or from TMA to TMAO in the intestinal bacteria, thus reduced blood TMAO. Mechanism study showed that BBR reduced TMAO production through inhibiting the activity of enzyme CutC and FMO of the gut microbiota. Biochemical analysis suggested that the mode of action of BBR on the bacterial enzyme CutC and FMO is closely related to its interaction with the CutC (radical form) and FMO coenzyme FAD (FAD-OOH), through its priority of transferring H^•^ from dhBBR to the CutC (radical form) or FAD-OOH, respectively. The mode of action of BBR on the intestine bacterial enzymes appears to be similar to that of vitamins. Clinical study showed that the average plaque score of 21 arteriosclerosis patients decreased by 3.2% after 4 months on BBR oral treatment, identifying BBR a potential drug to treat AS, at least partially, through inhibiting TMAO biosynthesis in the intestinal bacteria (Fig. 7).

Although the anti-AS effect of BBR might link to its systemic regulatory effect on lipid/glucose metabolism and inflammation, slowing-down TMAO production from choline in the gut microbiota might add new insight of the mechanism for BBR’s action against AS. In fact, through screening choline analogues Wang et al. found that 3, 3-dimethyl-1-butanol (DMB, an analogue of choline) had an obvious inhibitory effect on CutC, thus reduced the production of TMA; and they showed that DMB decreased the level of TMAO in animals.^16^ As TMA is metabolized into TMAO in human liver by FMO_3_, the transformation from TMA to TMAO has become a possible target against atherosclerosis as well.^17^ The present study showed that dhBBR, instead of BBR itself, might be the one that inhibits the activity of either CutC or FMO_._ DhBBR could reduce TMAO by down-regulating both choline-TMA and TMA-TMAO transformation through restricting CutC and FMO coenzymes (FAD) in intestinal bacteria, rather than inhibiting FMO_3_ in hepatocytes, as dhBBR in liver was almost undetectable.^37^ BBR treatment also showed inhibitory effect on TMA or TMAO production in vitro or in vivo, probably because of the BBR-dhBBR shift by NR (see below).

We have previously reported that BBR could be reduced into dhBBR in the presence of bacterial NR, and dhBBR is not stable and could be quickly oxidized back to BBR.^36,37^ We consider this shift between BBR and dhBBR very important for the action of BBR, with which both CutC and FMO activity could be down regulated through receiving the H^•^ from dhBBR. Due to the existence of 7-N lone pair electrons, the 8-C of dhBBR possesses H^•^ donating predisposition in the switch from dhBBR to BBR. In this case, the donation of H^•^ from dhBBR promotes the reaction between dhBBR and CutC (radical form) or FAD-OOH (FMO coenzyme), respectively. For details, dhBBR has priority to provide H^•^ for CutC (radical form) to form CutC (stable form), thus prevents CutC (radical form) from obtaining H^•^ from choline, leading to a reduction of choline cleavage and then TMA production. Similarly, as the electron delivery capability of dhBBR is stronger than that of TMA, the H^•^ provided by dhBBR could simply break the peroxide bond of FAD-OOH, thus generate FAD-OH and H_2_O,^47^ and prevent FAD-OOH from participating in TMA to TMAO transformation. As the function of both enzymes (and coenzymes) was down-regulated by dhBBR, TMAO production decreased in the intestine, and then declined in blood. This mode of action of BBR on the intestinal bacteria seems to be alike that of vitamins.

Vitamins are organic compounds essential for normal and healthy metabolism, although they are not an element of human body and do not provide energy. Vitamins are needed in small quantities and most of them need to be taken from food or medicine, as the human body either does not make enough of them, or it does not make any at all.^59^ Lack of vitamins might lead to abnormal health or even clinical diseases. The functions of a number of vitamins closely relate to enzyme catalysis, in which some vitamins are coenzymes or components of coenzymes.^59^ For instance, vitamin C (ascorbic acid) is a highly effective antioxidant, participating in many important biosynthesis processes in human body.^60^ Vitamin C has a 2, 3-ene-diol group which is easily dissociated to release hydrogen, making vitamin C a strong reducing agent, just like dhBBR in the intestine. Vitamin C in vivo is straightforwardly oxidized into dehydrogenated vitamin C, and the reaction is reversible and comparable to the switch between BBR and dhBBR in the intestine.^37^ As human body has no capacity to synthesize vitamin C, exogenous intake is essential. Vitamin C participates in the hydroxylation reaction that is necessary for the synthesis or decomposition of important substances, such as neurotransmitter and steroid.^61^ Besides, due to the reduction activity, vitamin C promotes the formation of tetrahydrofolate and activity of thiolase.^62^ Vitamin C could be either oxidized or reduced, and used as a hydrogen donor or a hydrogen acceptor; it plays an important role in the redox process in vivo.^63^ DhBBR, with similarity to vitamin C in some chemical properties, is also a strong reducing agent and tendentious to release hydrogen, showing important antioxidant ability, comparable to that of 2, 3-ene diol of vitamin C. The reducibility promotes the communication between dhBBR and CutC (radical form) or FAD-OOH (FMO coenzyme); at the same time, dhBBR is oxidized back to BBR. The switch of between BBR and dhBBR by intestinal bacteria NR and oxidases is comparable to that between vitamin C and dehydrogenated vitamin C. As dhBBR can’t be synthesized in body, it is provided by a NR-mediated reduction of BBR in food or medicine. Thus, dhBBR in the intestine could be a botanic agent with vitamin-like activity acting on intestinal bacteria. By principle however, BBR molecules that enter blood circulation should not follow this mechanism, as dhBBR is almost undetectable in blood and organs.^37^

For the clinical evaluation, these are several methods being used to evaluate the severity of atherosclerosis in clinic, including Doppler ultrasound, PET-CT, angioscopy, angiography et al.^64,65^. Doppler ultrasound is noninvasive, economical and relatively reliable to monitor carotid endarterectomy in different parts of blood vessels. It is the most commonly used method to evaluate the severity of atherosclerosis in clinical practice,^66^ and thus is the method used in the present study. To ensure the accuracy and reliability of BBR’s therapeutic efficacy, we examined every plaques of each patient at identical measurement spots before and after treatment, and assessed the progress of plaques in the patients in a comprehensive fashion. Internationally recognized plaque measurement indicators, such as common plaque score, carotid intima-media thickness as well as maximum plaque length were employed in the clinical study to analyse the drug efficacy.^56^ Using the measurements, BBR’s therapeutic efficacy was examined in the patient cohort. Although the baseline values (such as plaque score, glucose or TG level) in the group of statin plus anticoagulants were slightly higher than those in the BBR’s group, which might be a limitation in evaluating the therapeutic efficacy for this cohort, the plaque score of 21 AS patients in BBR’s group (treated for 4 months) was reduced by 3.2% (*P < 0.05), demonstrating a therapeutic outcome, at least similar to that of combination therapy using statin and anticoagulant. All participants in the BBR group showed good tolerance and safety in the whole treatment course.

Atherosclerosis is very difficult to treat once the plaque forms. Currently, the main drug regimen is the combination of statins with anticoagulants drugs, such as aspirin,^57^ but the clinical efficacy of the treatment is not satisfactory, and their side effect is a concern.^67,68^ Thus, safe and effective anti-atherosclerotic drugs are highly desirable. We consider the presented BBR results from AS patients of significance for future treatment and prevention of plaque formation in clinical practice. In fact, BBR treats metabolic disorders through multiple biological targets in body as well as in intestinal bacteria,^9,26,29,30,69^ thus it implements a synergistic drug Cloud effect, including reduction of TMAO level in the treatment of atherosclerosis.^29^ Apparently, large-scale clinical studies are needed to draw conclusion of using BBR to treat AS.

## Conclusion

In summary, oral BBR reduced TMAO production in the gut microbiota, followed by a decrease of TMAO in blood. This effect might be an important part of its action against plaque in AS. The TMAO-lowering effect by BBR in gut flora attributes to its inhibitory activity on enzyme CutC and FMO of bacteria. The mode of action suggests a vitamin-like mechanism of BBR on intestinal bacteria. BBR-caused TMAO reduction was seen in the AS patients, in whom BBR’s therapeutic effect against plaque was observed. Thus, it is justified to develop BBR as a medicine for the treatment of atherosclerosis.

## Materials and methods

### Animals

SD rats (male, 180–220 g) and hamsters (Mesocricetus auratus, male, 8 weeks, 100–120 g) were supplied by the Vital River Laboratory Animal Technology Co., Ltd. (Beijing, China). Animals were housed in a controlled environment with free access to food and water. The room temperature was maintained at 22 ± 2 °C with a 12-h light/dark cycle. The research was conducted in accordance with the institutional guidelines and ethics and was approved by the Laboratories’ Institutional Animal Care and Use Committee of the Chinese Academy of Medical Sciences and Peking Union Medical College (No. 00001025, 00005409, 00001024). The research was conducted in accordance with all guidelines and ethics of the Chinese Council on Animal Care.

### Efficacy of BBR on the plaques of hamsters with arteriosclerosis

Thirty-seven hamsters (male, 8 weeks, 100–120 g) were adapted for a week before grouping. Then, eight hamsters were fed with the regular fodder (the normal control group, Group N), and the other twenty-nine hamsters had free access to HFD for ten months to establish the atherosclerosis model. All the atherosclerotic hamsters were randomly divided into five groups and given different diets or drugs for three months. The five groups were the atherosclerosis model group (Group H, *n* = 7), the low-dosage BBR group (oral, 100 mg/kg/d, Group BL, *n* = 7), the high-dosage BBR group (oral, 200 mg/kg/d, as Group BH, *n* = 5), the antibiotics group (oral, terramycin 300 mg/kg/d, erythromycin 300 mg/kg/d and cefadroxil 100 mg/kg/d, Group A, *n* = 5) and the antibiotics plus BBR group (oral, terramycin 300 mg/kg/d, erythromycin 300 mg/kg/d, cefadroxil 100 mg/kg/d, and BBR 200 mg/kg/d, Group AB, *n* = 5). In the BH group, one animal died due to its own reasons before the end of treatment, so the corresponding data could not be collected. Therefore, the number of animals used for statistics in BH group was four. Levels of FBG and lipids (TG, TC, and LDL-C) were measured with commercial kits (BioSino Bio-Technology & Science Inc., Beijing, China). At the end of the 3-month treatment, the maximum intima-media thickness (IMT_max_) of the aortic arch in all the hamsters was determined by ultrasonic imaging. After scarification, the aortic arch of hamsters was collected for morphological imaging and histologic assessment, including oil red O and hematoxylin and eosin (HE) straining. TMA/TMAO/choline level in faeces or blood in the hamsters was detected using LC-MS/MS 8050.

### Clinical trial of BBR treatment

To assess the therapeutic efficacy of BBR on atherosclerosis, 37 individuals were randomly enrolled in the Outpatient Section of the First Hospital of the Jilin University in Changchun The study was approved by the institutional ethics committee of the hospital (clinical study No. 2017-251-1) and registered in the China Clinical Trial Registry (ChiCTR-OPN-17012942). Of the 37 individuals, 16 subjects (Group 1; 7 males, 9 females; age 60.5 ± 8.9) had their blood lipid and glucose levels in the normal range [TC (mmol/L), 4.59 ± 0.57; TG (mmol/L), 1.12 ± 0.37; LDL-C (mmol/L), 2.49 ± 0.38; FBG (mmol/L), 5.16 ± 0.49]. The remaining 21 individuals (Group 2, 12 males, 9 females; age 63.9 ± 5.4) had high levels of glucose or lipids in blood [TC (mmol/L), 5.70 ± 1.04; TG (mmol/L), 3.65 ± 4.55; LDL-C (mmol/L), 3.11 ± 0.85; FBG (mmol/L), 6.79 ± 2.29]. In the Group 2, the 21 patients had been diagnosed with atherosclerosis by the Doppler ultrasonography (Delica Medical Equipment Co., Ltd., Shenzhen, China). These subjects with atherosclerosis were not undergoing any drug treatment before enrolment. Serum and faecal samples were obtained from all of the individuals before treatment. After informed consents were obtained, the subjects in Group 2 were treated with BBR (oral, 0.5 g, bid; Beijing Zhongxin Pharmaceutical Co. LTD, Beijing, China) for 4 months.

### Statistical analysis

The statistical analyses were performed by the two-tailed Student’s *t* test or paired *t* test using the GraphPad Prism Version 5 (GraphPad Software, CA, USA). All of the data are presented as the mean ± standard deviation (SD) or standard error (SEM), and *P* values less than 0.05 were considered statistically significant.

## Supplementary information

Supplementary Materials for Berberine treats atherosclerosis via a vitamine-like effect down-regulating Choline-TMA-TMAO production pathway in gut microbiota

## Data Availability

The data used and/or analyzed to support the findings of this study are available in this paper or the Supplementary Information. Any other raw data that support the findings of this study are available from the corresponding author upon reasonable request.
