# Supplementary material for: Berberine treats atherosclerosis via a vitamine-like effect down-regulating Choline-TMA-TMAO production pathway in gut microbiota
Source: Signal Transduct Target Ther. 2022 Jul 7;7:207. doi: 10.1038/s41392-022-01027-6 (PMC9259588; doi:10.1038/s41392-022-01027-6)
Supplement: Supplementary file 1 — Supplementary Materials for Berberine treats atherosclerosis via a vitamine-like effect down-regulating Choline-TMA-TMAO production pathway in gut microbiota [file 41392_2022_1027_MOESM1_ESM.pdf]

Supplementary Materials for

Berberine treats atherosclerosis via a vitamine-like effect down-regulating Choline-TMA-TMAO production pathway in gut microbiota

Shu-Rong Ma[1,#], Qian Tong[2,#], Yuan Lin[1,#], Li-Bin Pan[1,#], Jie Fu[1,#], Ran Peng[1] , Xian-Feng Zhang[2], Zhen-Xiong Zhao[1], Yang Li[2], Jin-Bo Yu[1], Lin Cong[1], Pei Han[1], Zheng-Wei Zhang[1], Hang Yu[1], Yan Wang[1, *], Jian-Dong Jiang[1, *]

[1] State Key Laboratory of Bioactive Substance and Function of Natural Medicines, Institute of Materia Medica, Chinese Academy of Medical Sciences/Peking Union Medical College, Beijing 100050, China

[2]The First Hospital of Jilin University, Changchun 130021, China

[#] These authors contributed in equal.

* Corresponding author: Dr. Yan Wang, Tel.: +86 10 63165238, fax: +86 10 63165238, e-mail address: wangyan@imm.ac.cn; or Dr. Jian-Dong Jiang, Tel.: +86 10 83160005, fax: +86 10 63017757, e-mail address: jiang.jdong@163.com.

This PDF file includes:

Supplementary Materials & Methods

Figures S1 to S10

Tables S1 to S11

Supplementary Materials & Methods

Chemicals and reagents

Berberine hydrochloride (BBR), trimethylamine hydrochloride, and benzylamine (internal standard, IS) were purchased from J&K Scientific Ltd. (Beijing, China). Trimethylamine-n-oxide was obtained from Tokyo Chemical Industry Co., Ltd (Tokyo, Japan). Imipramine, methimazole, choline and carnitine were acquired from Solarbio Life Science Co., Ltd. (Beijing, China). 3, 3-Dimethyl-1-butanol (DMB) was obtained from Sigma-Aldrich (Darmstadt, Germany). Dihydroberberine (dhBBR) and tetrahydropalmatin were purchased from Chengdu Must Bio-technology Co., Ltd. (Chengdu, China). The purity of all the compounds was above 98%. HPLC-grade acetonitrile and methanol were obtained from the Fisher Scientific (Fair Lawn, USA). Deionized distilled water was obtained from the Hangzhou Wahaha Group Co. Ltd. (Hangzhou, China). All the other chemicals and reagents were obtained from the Sinopharm Chemical Reagent Co., Ltd. (Beijing, China). Assay kits for fasting blood glucose (FBG), total cholesterol (TC), low-density lipoprotein cholesterol (LDL-C) and triglycerides (TG) were from the BioSino Bio-technology & Science Inc. (Beijing, China).

BBR analysis in blood

The concentration of BBR in the plasma of the HFD-fed hamsters was determined by LC-MS/MS 8050 as described before.[35,39,42]

Instruments for TMA/TMAO detection

Liquid chromatography with tandem mass spectrometry (LC-MS/MS 8050, Shimadzu Corporation, Kyoto, Japan) was used for the quantification analysis of TMA and TMAO. LC separation was achieved using an Alltima C_{18} column (5 μm x 250 mm, W. R. Grace & Co, Columbia, USA). The mobile phase was composed of water-formic acid (100:0.2, v/v) (A) and acetonitrile (B) with a linear gradient elution (A:B, 0.01 min, 80:20; 5.00 min, 80:20; 5.01 min, 5:95; 7.00 min, 5:95; 7.01 min, 80:20; 11.00 min controller stop) at a flow rate of 0.6 mL/min (30 °C). Shimadzu LCMS Solution (Version 5.72) was used for data acquisition and processing. The mass parameters in positive mode (ESI) were set as the following: nebulizer gas, 3 L/min; drying gas, 10.0 L/min; interface, -4.5 kV; CID gas, 230 kPa; DL temperature and heat block temperature at 200 and 250 ℃, respectively. The multiple reaction monitoring (MRM) parameters were as follows:

60.20→44.00 (m/z) for TMA, 76.15→58.05 (m/z) for TMAO and 108.20→91.10 (m/z) for benzylamine (IS). The method validation assays (including hamster plasma, hamster faeces and culture medium as the different matrix, respectively) were carried out according to the currently accepted bioanalytical method validation guidelines, and the validation contents included selectivity, linearity, precision, accuracy, extraction recovery, matrix effect and stability. Apart from this, to avoid the possible interferences produced by structurally similar substances like choline or carnitine, the measurement was further validated by addition of choline or carnitine (1 μg/mL).We compared the quantification results of TMA and TMAO before and after choline or carnitine added, respectively, with CV values [coefficient of variation, CV (%) = (SD/Mean) × 100%] less than 15% as the accepted limit.

In addition, a published method with isotope-labelled internal standard (IS) [42] was to verify the reliability of the current LC-MS/MS method (with benzylamine as internal standard) for TMA/TMAO measurement. CV values of TMA/TMAO by two methods were calculated through determination of three practical plasma and faecal samples respectively. Briefly, as described in the published method [42], d9-TMA (Sigma-Aldrich, Munich, Germany) and d9-TMAO (Sigma-Aldrich, Munich, Germany) were used as the internal standards. Firstly, 100 μL plasma or diluted faecal samples were treated with 300 μL of cold internal standard mix (10 μmol/L of d9-TMA and d9-TMAO in methanol/acetonitrile=15:85 and 0.2% formic acid) to precipitate the protein, followed by centrifugation at 10,000 ×*g* for 5 min and transferred to sample vials for LC-MS/MS. The chromatographic separation was performed using an Acquity UPLC BEH HILIC column (1.7 μm×2.1 mm×100 mm, Waters, Milford, USA) with an Acquity HILIC VanGuard precolumn (1.7 μm×2.1 mm×5 mm, Waters, Milford, USA). The mobile phase consisted of 15 mmol/L ammonium formate (pH 3.5) as solvent A and acetonitrile as solvent B, with the gradient conditions: 0.0–2.0 min, 10–30% A; 2.0–3.5 min, 30% A; 3.6–4.6 min, 40% A; and 4.7–6.0 min, 10% A. The flow rate was kept at 0.4 mL/min and 3 μL was injected to the LC-MS/MS system. The multiple reaction monitoring (MRM) parameters were as follows: 60.2→44.0 (m/z) for TMA, 76.0→58.0 (m/z) for TMAO, 69.2→49.0 (m/z) for d9-TMA (IS) and 85.0→66.0 (m/z) for d9-TMAO (IS). The detection was performed on LC-MS/MS 8050 (Shimadzu Corporation, Kyoto, Japan). The mass parameters in positive mode (ESI) were set as the following: nebulizer gas, 3 L/min; drying gas, 10.0 L/min; interface, -4.5 kV; CID gas, 230 kPa; DL temperature and heat block temperature at 250 and 300 ℃, respectively.

TMA or TMAO in plasma and faeces in hamsters treated with BBR

Eighteen hamsters (8 weeks old) were randomly separated into three groups (6 for each). Twelve hamsters were fed with special fodder (83.9% basic diet, 15.0% fat lard, 1.0% cholesterol and 0.1% sodium cholate) for 2 months to establish the hyperlipidaemia model, and the other six were fed with regular diets. Then, the HFD hamsters entered experiment course. Among them, six hamsters were treated with BBR orally (100 mg/kg) (Group 1), and the other six were intraperitoneally administrated with BBR (20 mg/kg) (Group 2). The last six hamsters fed regular diets were orally treated with BBR (100 mg/kg) (Group 3). Plasma and faecal samples were obtained at 0, 6, 12 and 24 h after BBR administration. Approximately 0.2 grams of faecal samples were thoroughly mixed with formic acid: water (0.2: 100) solution at 1: 4 [weight (g): volume (mL)] followed by centrifugation at 12,000 g for 5 min (4 ℃), then the supernatant was collected. Next, 150 μL acetonitrile (with 0.2% formic acid and 25 ng/mL benzylamine) was added to 50 μL of plasma or pretreated faecal sample to precipitate the protein.[42] After mixing and centrifugation at 12, 000 × *g* for 5 min, 10 μL of supernatant was injected for TMA/TMAO analysis by LC-MS/MS 8050. The series working solutions were prepared as 500, 200, 100, 20, 10, 5, 2, 1, and 0.2 ng/mL by diluting the stock solution (with 0.2% formic acid-water) at 4 ℃ and pre-treated with acetonitrile as above.

TMA or TMAO in plasma and faeces of hamsters treated with saline

To exclude the difference in circadian rhythm, another six HFD hamsters were treated with saline orally (0.5 mL) and plasma and faecal samples were obtained at 0, 6, 12 and 24 h after the saline administration. Then, 50 μL of plasma or pretreated faecal samples were added with 150 μL of acetonitrile (with 0.2% formic acid and 25 ng/mL benzylamine) to precipitate the protein. After mixing and centrifugation at 12, 000 × *g* for 5 min, the supernatant was injected for TMA/TMAO analysis by LC-MS/MS.

Fecal microbiota transplantation experiment

For fecal transplantation experiment, 30 hamsters (8 weeks, male) were randomly divided into five groups: Group 1, the normal control group (Normal group); Group 2, the high-fat diet group (HFD group); Group 3, the high-fat diet group treated with BBR (oral, 200 mg/kg) (HFD+BBR group); Group 4, the fecal transplantation group treated with fecal bacteria from Group 2 [HFD+FT(HFD,Group2)]; Group 5, the fecal

transplantation group treated with fecal bacteria from Group 3 [HFD+FT(HFD+BBR, Group3]. After adapting for 3 days, animals of Group 1 received the regular fodder during all the experiment, and animals of Group 2 received the high-fat diet for modeling the hyperlipidemia. After day 15, hamsters of Group 2 were orally treated with BBR at 200 mg/kg until the end of the experiment. After day 30, the fresh fecal samples from Group 2 and Group 3 were collected, thoroughly mixed with PBS (weight: volume=1:9) and filtered to prepare the bacterial solution. And then, the bacterial solution was administrated to hamsters of Group 4 and Group 5, respectively (once a day, 0.5 mL/100g, for 10 days). After fecal transplantation, the fecal and plasma samples of Group 1 to 5 were collected and analyzed for TMA and TMAO levels.

TMA or TMAO in the gut microbiota after incubating with BBR or dhBBR

Colon contents of rats were collected. Approximately 5 grams of the pooled content mixture was transferred into a sterile flask and mixed with anaerobic culture medium (Solarbio Life Science Co., Ltd, Beijing, China) at a ratio [weight (g): volume (mL)] of 1:20. After mixing thoroughly and filtering, the intestinal bacteria culture was pre-incubated under anaerobic conditions (N_2 atmosphere) at 37 ℃ for 60 min. Next, 10 μL BBR or dhBBR solution (3 mM and 6 mM) was added to the bacterial culture (990 μL) under anaerobic conditions, with dimethylsulfoxide (DMSO, 10 μL) added as a negative control. The incubation was at 37 ℃ for 6, 12 and 24 h, separately, and then 100 μL of the culture was processed by adding 300 μL of acetonitrile (with 0.2% formic acid and 25 ng/mL benzylamine) for TMA and TMAO analysis.

Stability of TMA/TMAO and BBR/dhBBR in medium

TMA, TMAO, BBR or dhBBR in the anaerobic culture medium (Solarbio Life Science Co., Ltd, Beijing, China) were incubated for 24 h under anaerobic condition at 37 ℃. 10 μL of the compounds (10 mg/mL TMA, TMAO, BBR or dhBBR) was added to 990 μL of the culture and samples were collected at 2, 4, 6, 8, 12, 24 h, separately. Then 100 μL of the culture was processed for analysis.

TMA in the gut microbiota after treated with study agents *in vitro*

10 μL of study agents (BBR, dhBBR or DMB) was mixed with 990 μL of the intestinal bacterial culture solution from HFD rats. The final concentration was 0.03 and 0.06 mM for BBR or dhBBR, and 0.12, 0.3 mM for DMB. After 12 h incubation in the anaerobic

conditions at 37 ℃, the samples were pre-treated as described above and analysed for the TMA detection

Additionally, dhBBR (0.06 mM) was added into the gut microbiota from HFD-SD rats and incubated for 0, 2, 6, and 12 h, with identical volume of DMSO added as control. Then, 50 μL of the mixed culture was pre-treated by three folds of acetonitrile (with 0.2% formic acid and 400 ng/mL benzylamine) for choline analysis. The quantitative parameter (MRM mode) for choline was 103.95→60.05 (m/z) by LC-MS/MS 8050.

TMA in the gut microbiota after incubating with choline or carnitine

Choline or carnitine, dissolved in sterile saline, was added to the bacterial culture under the aseptic operation with the final concentration of 40 μg/mL. After incubation at 37 °C for 12 h, the intestinal bacterial culture samples were prepared for chemical analysis as described above. TMA level was tested by LC-MS/MS 8050. Additionally, a heat-inactivated bacterial culture samples were prepared as the negative control.

Molecular docking between BBR/dhBBR and enzymes (CutC, CCAT and FMO)

The possible interactions between BBR or dhBBR and enzymes were calculated by CDOCKER algorithm in the drug-receptor interactions mode using Discovery Studio Client software (v16.1.0.15350). The crystal structure of bacterial FMO was available in the Protein Data Bank (DOI: 10.2210/pdb2VQ7/pdb). The binding site was selected automatically from the receptor cavities. The parameters were default values, except that the pose cluster radius was set at 0.5. The crystal structure of bacterial CutC and CCAT was available in the Protein Data Bank. The interactions between the drug compound (dhBBR or BBR) and CutC (DOI: 10.2210/pdb5FAU/pdb) or CCAT (DOI: 10.2210/pdb1XA4/pdb) were performed as described above.

TMA / TMAO in 15 individual strains of the intestinal bacteria *in vitro*

Fifteen individual bacteria strains, which are abundant in human gut microbiota, were used to investigate TMA and TMAO biosynthesis after BBR treatment *in vitro*. The bacterial strain of *Proteus mirabilis* (*P. mirabilis,* ATCC35659), *Enterobacter cloacae* (*E. cloacae,* ATCC700323), *Peptostreptococcus anaerobius* (*P. anaerobius,* ATCC27337), *Enterobacter aerogenes* (*E. aerogenes*, ATCC13048), *Escherichia coli* (*E. coli,* ATCC25922), *Enterococcus faecium* (*E. faecium*, ATCC35667), *Shigella boydii* (*S. boydii*, CMCC51522), *Staphylococcus epidermidis* (*S. epidermidis,* ATCC12228),

Klebsiella pneumoniae (*K. pneumonia*, ATCC70603), *Staphylococcus aureus* (*S. aureus*, ATCC6538), *Lactobacillus acidophilus* (*L. acidophilus*, ATCC314), *Bacteroides fragilis* (*B. fragilis*, ATCC25285), *Bifidobacterium longum* (*B. longum*, CICC6068), *Bifidobacterium breve (B. breve*, ATCC15700) and *Pseudomonas aeruginosa* (*P. aeruginosa*, PAO1) were obtained from Nanjing Bianzhen Biotechnology Co. LTD (Nanjing, China). The bacterial strains of *P. mirabilis*, *E. aerogenes*, *Escherichia coli*, *S. epidermidis*, *S. aureus*, and *P. aeruginosa* were cultured in Luria-Bertani medium. *E. cloacae*, *P. anaerobius*, *E. faecium*, *S. boydii*, *K. pneumonia*, *B. fragilis* and *L. acidophilus* were cultured in MRS broth medium. *B. longum* and *B. breve* were cultured in 0.5% Cysteine modified MRS medium. After activation and unification at 3×10^8 CFU/mL, bacterial strains were incubated with BBR (0.03 mM) in the anaerobic condition at 37 ℃ for 12 h, with an identical volume of methanol added as the negative control. After incubation, 500 μL of the culture samples were diluted proportionally, and the colony numbers were determined under 600 nm by an ultraviolet spectrophotometer (Shimadzu Corporation, Kyoto, Japan). 100 μL of the resulting sample was prepared for TMA and TMAO analysis by LC-MS/MS 8050.

TMA in *P. mirabilis* / *P. aeruginosa in vitro*

Powdered *P. mirabilis* or *P. aeruginosa* was revived overnight and amplified under anaerobic conditions at 37 ℃. After monitoring at OD_{600nm} value and diluting to 3×10^8 CFU/mL, the bacterial culture (990 μL) was treated with 10 μL of BBR or dhBBR or DMB at a final concentration of 0.03 mM, 0.03 mM, 0.1 mM, respectively, then incubated at 37 °C for 12 h. 100 μL resulting sample was for TMA analysis with LC-MS/MS 8050. Colony numbers were determined by OD_{600nm} after incubation.

Plasmid construction and function verification of CutC

Active CutC enzyme consists of two parts, choline trimethylamine-lyase (gene: *cutC*) and its activase (gene: *cutD*). As *cutC* existed in *E. coli* BL21, *cutD* (AM942759.1) was amplified with the following primers (Pmtclo-F ， TGGGTCGCGGATCCGAATTCATG GAGACGGCAGCAGAAATAA and Pmtclo-R， TCGAGTGCGGCCGCAAGCTTTTAGTGGCG GACAAGACGGA）, using genomic DNA of *P. mirabilis* as a template by PCR. Then, the gene fragment *cutD* was cloned

into the HindIII/EcoRI sites of expression vector pET28a to construct the recombinant *pET-cutD* vector. DH5α *E. coli* cells were transformed by a heat shock method and used for plasmid propagation. Before expression, the *cutD* positive clone was confirmed by sequencing. Then protein CutD (WP_004249185.1) expression was conducted using *E. coli* BL21 (DE3). Cells were grown in liquid LB media supplemented with kanamycin (40 μg /mL). Cultures were maintained at 37 °C until OD_{600nm} reached 0.6. Then, the temperature was lowered to 16 ℃ and the protein expression was induced with 1.0 mM isopropyl β-D-1-thiogalactopyranoside (IPTG) for 20 h. The bacterial cells were harvested by centrifugation (10, 000 ×g, 20 min, 4 ℃), 20 times concentrated and suspended in about 10 mL of PBS solution. After ultrasonic decomposition, the supernatant and precipitate were prepared for SDS-PAGE analysis with the blank pET28a plasmid in *E.coli* BL21 cells as control. After concentration of the supernatant by microporous membrane (MW: 10,000) to 500 μL, the enzyme lysate with CutC/CutD was used for functional verification under anaerobic conditions. The enzyme reaction system (100 μL) consisted of CutC *E. coli* (pET28a), with or without CutD (10 μL), and the choline substrate (0.1 mM) in PBS solution. DhBBR (0.06 mM) was additionally added in the reaction system of CutC/CutD *E. coli* (pET28a) supernatant for inhibition study. The incubation time was 8 h, followed by TMA measurement using LC-MS/MS as described.

TMAO in the gut microbiota after incubating with BBR or dhBBR *in vitro*

The intestinal bacteria (from the regular diet-fed SD rats) culture solution was prepared as described above. Then, 10 μL of BBR or dhBBR was added to the 990 μL of the bacterial culture solution to obtain a final concentration of 0.03 and 0.06 mM. Next, the culture was incubated at 37 ℃ under anaerobic conditions for 6 h， in order to analysing TMAO levels. The known FMO inhibitor imipramine (0.03, 0.06 mM) and methimazole (0.1 and 0.33 mM) were used as positive controls.

TMAO in *P. aeruginosa* treated with study agents *in vitro*

P. aeruginosa powder was revived overnight and amplified in the corresponding liquid LB medium (Solarbio Life Science Co., Ltd, Beijing, China) under anaerobic conditions. The number of colonies in medium was measured by OD_{600nm} and diluted to 3 × 10^8 CFU/mL. 10 μL of BBR or dhBBR (3 mM) or imipramine (10 mM, the inhibitor of FMO) was added to 990 μL of bacterial culture to assess TMAO levels after the 12 h incubation

(37 ℃, anaerobic conditions). The final concentration of BBR, dhBBR and imipramine in the incubation was of 0.03, 0.03, 0.1 mM, respectively. Next, 10 μL of DMSO was added to an identical volume of bacterial culture as control. A total of 100 μL of the resulting samples was mixed with 3 volumes of acetonitrile (with 0.2% formic acid and 25 ng/mL benzylamine), followed by centrifugation for 5 min. The supernatant was ready for TMAO analysis using method described above. Colony numbers were determined by OD_{600nm} after incubation.

Cloning of the *fmo* gene and plasmid construction

The chromosomal DNA of *P. aeruginosa* PAO1 was extracted and purified using TIANamp Bacteria DNA kit (Tiangen Biochemical Technology Co., Ltd., Beijing, China) according to the instructions. The gene *fmo* (NC_022591.1) was amplified using the primers -F/ -R. Primers for the *fmo* gene were designed by primer premier 5.0 as follows: -F: AGCAAATGGGTCGCGGATCCGTGTATACGCCAGCAAACAATCA; -R: TCGAGTGCGGCCGCAAGCTTTCATGCGGGTACCCCTTCG. Polymerase chain reaction was performed for 35 cycles in a 50-μL system with phanta polymerase (Novizan, China). Then, 1% agarose gel electrophoresis and gene sequencing were conducted to verify the gene. The *fmo* fragment was cloned into the HindIII/BamHI sites of the expression vector pET28a (Invitrogen, USA) to obtain pET28a-*fmo,* using Gibson Assembly Master Mix (NEB, USA).

Expression of the *fmo* gene and function verification

The plasmid pET28a-*fmo* was transformed into *E. coli* BL21 cells (Novizan, China). After verifying the sequence, the clones were used to express the FMO protein (WP_003109151.1). The *E. coli* BL21 colony containing pET28a-*fmo* was grown in 100 mL LB medium with 15 μg/mL kanamycin at 37 ℃. When the amplified bacteria reached 0.6-0.8 under OD_{600nm}, isopropyl β-D-1-thiogalactopyranoside was added at a final concentration of 0.1 mM to induce expression of target protein at 16 ℃ for 20 h. The bacterial cells were harvested by centrifugation, 20 times concentrated and suspended in about 10 mL of PBS solution, and after ultrasonic decomposition, the supernatant and precipitate were prepared for SDS-PAGE electrophoresis, with blank pET28a plasmid in *E.coli* BL21 cells (free of *fmo*) as control. After concentration of the supernatant by microporous membrane (MW: 30,000) to 500 μL, functional verification of FMO was performed using the concentrated enzyme lysate.

The enzyme reaction system (100 μL) consisted of NADPH (0.1 mM), flavin adenine dinucleotide (0.1 mM), the enzyme lysate (with pET28a or pET28a-*fmo* plasmid, 20 μL), and the TMA substrate (0.1 mM) in PBS solution. BBR or dhBBR (0.06 mM) was added, followed by incubation for 4 h. Then, the product was ready for analysing TMAO production, using LC-MS/MS as described above.

Effect of dhBBR in the FMO reaction systems

Effect of dhBBR on FMO was examined in the enzyme reaction system as described above. The dose of dhBBR in the reaction system was as following: 0.0075, 0.015, 0.03, 0.06, 0.24 mM, respectively, and the systems were incubated for 4 h. Then, cold acetonitrile with internal standard was added immediately for TMAO analysis.

At the same time, dhBBR (0.06 mM) was introduced into the FMO reaction system and culturing for 0, 2, 4, and 8 h. Then, the resulting solutions were divided into two parts (50 μL for each). 50 μL of solution was pre-treated with cold acetonitrile (150 μL, with 0.2% formic acid and 25 ng/mL benzylamine) and prepared for the analysis of TMA and TMAO. Other 50 μL was added to 200 μL of methanol and 10 μL of tetrahydropalmatin (IS, 400 ng/mL) to determine the concentration of BBR as described above.

Effect of BBR or dhBBR on TMAO in the liver homogenate

After sacrifice by cervical dislocation and heart perfusion, the liver tissue was collected from six SD rats. Saline was added [weight (g): volume (mL) = 1: 4] at 4 ℃ and the liver homogenate was prepared with a homogenizer (IKA®-Werke GmbH & CO. KG, Staufen, Germany). Next, BBR, dhBBR or imipramine was cultured with the liver homogenate (at concentrations of 0.06 and 0.12 mM) for 2 h after mixing. Meanwhile, methanol was added to the homogenate as the control. The TMAO level was determined with LC-MS/MS 8050.

FMO level in atherosclerotic hamsters

The ELISA kit of FMO was obtained from JiangLai Biological Technology Co., Ltd (Shanghai, China). The liver tissue was firstly mixed with 9 fold of PBS solution (weight: volumn=1: 9) for homogenate by homogenizer (IKA Works GmbH & Co., Staufen, Germany) on ice. Then, the mixture was ultrasonic breaking for 30 min (every five seconds of ultrasound with 10 seconds interval). Then, the liver homogenate was

centrifuged at 5000 rpm for 5 min, the supernatant was collected for analysis. And the measurement procedure was followed by the ELISA instrument.

qPCR of 16s rDNA gene in hamster feces

The plasmid DNA of fecal bacteria in atherosclerotic hamsters was extracted by kits (DP105, TIANGEN, China). Group N, the normal control group; Group H, the atherosclerosis model group fed with HFD; Group BL, the low-dosage BBR group (oral, 100 mg/kg/d); Group BH, the high-dosage BBR group (oral, 200 mg/kg/d); Group A, the group treated with antibiotics; Group AB, the group treated with BBR and the combination of antibiotics (oral, terramycin 300 mg/kg/d, erythromycin 300 mg/kg/d, cefadroxil 100 mg/kg/d, and BBR 200 mg/kg/d; 3 months). Then, OD_{260nm} were monitored by micronucleic acid quantifier. The 16s rDNA gene copies in atherosclerotic hamster feces were carried out by qPCR (Fast qPCR Mix, SYBR Green I). The primers are as following: -F, ACTCCTACGGGAGGCAGCAG; -R, GGACTACHVGGGTWTCTAAT. The total volume of reaction was 20 μL and included 40 cycles. Then, threshold cycle (Ct) was recorded for the quantification of gene copies in samples.

Bacterial composition analysis

Faecal samples of animals or clinical subjects were collected for bacterial composition analysis as previously described.[39] After DNA was extracted (Omega Bio-Tek, Norcross, GA, USA), the specific primers 340F and 805R targeting the V3-V4 region of the gut bacterial 16S rRNA gene were used for amplification. The product mixture was purified (GeneJET Gel Extraction Kit, QIAGEN, Germany) and prepared for sequencing (NEXTflex Rapid Illumina DNA-Seq Kit, Ipswich, MA). The HiSeq 2500 platform (Illumina, San Diego, USA) was used for sequencing the library, and 250-bp paired-end reads were obtained. After categorizing the reads, each operational taxonomic unit (OTU) with a similarity over 97% was annotated for classification by the ribosomal database. Species counts of the animal faeces were performed by QIIME version v.1.8. (Evanston, USA).

Clinical trial of BBR treatment

Serum samples from 37 individuals were collected and pre-treated immediately for blood glucose or lipid analysis; faeces or the remaining blood samples were placed

directly at -70 ℃. The results of TC, LDL-C, TG, and FBG in blood were provided by the clinical laboratory of the First Hospital of Jilin University. The plaque morphology of atherosclerosis of the subjects (Group 2) was examined and recorded by Doppler ultrasonography before and after 4 months BBR treatment. The sites of examination included 12 locations, namely, bilateral common carotid artery, bilateral carotid bifurcation, bilateral internal carotid artery, bilateral external carotid artery, bilateral vertebral artery intervertebral space and bilateral subclavian artery. The calculation to quantify the severity of plaques were as following: 1) plaque score (mm), the sum of the thickness of the plaques per patient, and the thickness of each plaque was recorded as Dist A value by Doppler ultrasonography; 2) carotid intima-media thickness (mm), the thickness of each plaque, which was identical with Dist A result by Doppler ultrasonography; 3) carotid plaque length (mm)， the determined length of each plaque of patients on the cross section of vessel. Apart from this, bilateral brachial-ankle pulse wave velocity value was determined to access the risks of plaques. One gram of faecal sample of the subjects (Group 1 and Group 2) before and after BBR treatment was collected for bacterial composition analysis. TMA/TMAO levels in faeces or serum of the study subjects, before and after BBR treatment, were detected by LC-MS/MS 8050.

As a reference group, 12 atherosclerosis subjects treated with known drugs were enrolled (Group 3) in the study. All of the patients (10 males and 2 females; age 55.6 ± 8.9) had been diagnosed with atherosclerosis and showed abnormal levels of glucose or lipids before enrolment [TC (mmol/L), 4.53 ± 1.10; TG (mmol/L), 1.54 ± 0.51; LDL-C (mmol/L), 2.77 ± 0.73; FBG (mmol/L), 7.71 ± 3.44]. All the patients underwent conventional therapy according to the CVD treatment guidelines in the European Society of Cardiology and World Heart Federation. They provided informed consent and agreed that their personal data could be used in scientific research. After ruling out contraindications for statins, the 12 patients were prescribed with rosuvastatin calcium (Astra Zeneca, China) and aspirin protect tablets (Bayer, China), as well as clopidogrel sulfate (SANOFI, China) or ticagrelor (Astra Zeneca, China) according to their symptoms. The morphology of atherosclerotic plaque in subjects was recorded by ultrasonography before and after 4 months of treatment at the identical examining sites.

Supplementary Figures

Fig. S1

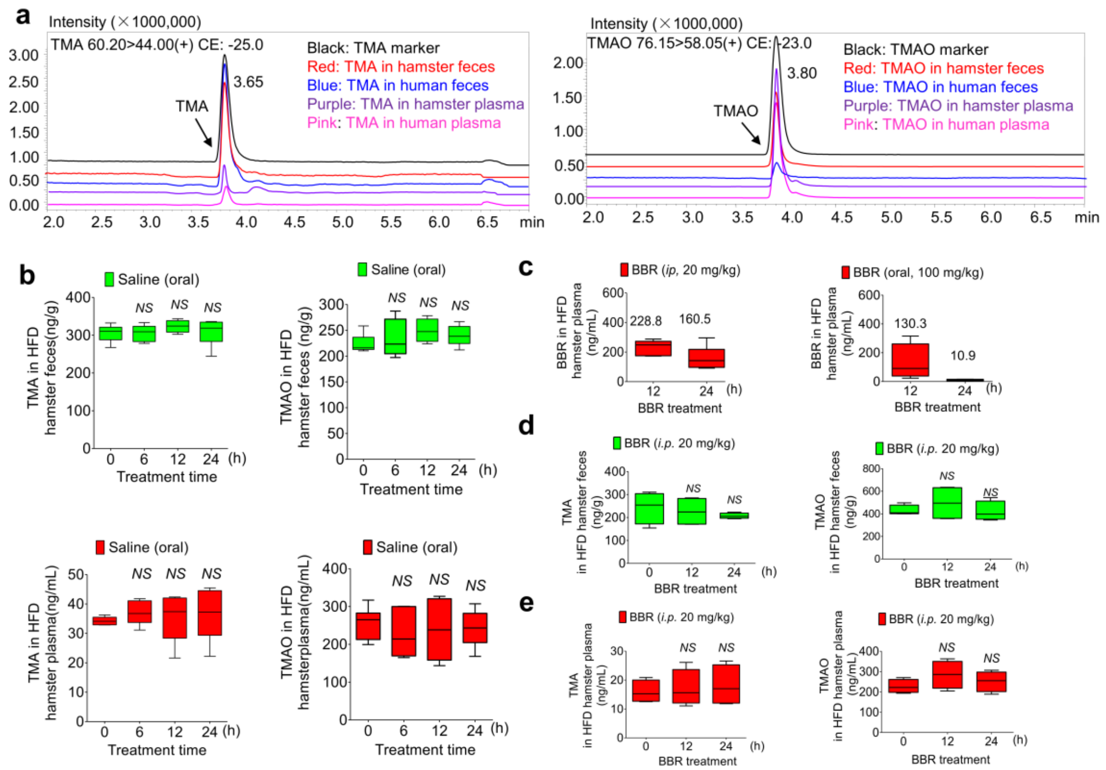

Fig. S1 TMAO levels were not decreased after oral administration of saline or intraperitoneal injection of BBR.

a The mass spectra of TMA and TMAO in faeces/plasma samples of hamsters and humans. **b** The levels of TMA and TMAO in faeces or plasma of the HFD-fed hamsters were not changed at 6, 12, and 24 h after oral administration of saline (0.5 mL, n=6, *NS*, not significance). **c** Plasma BBR level in the HFD-fed hamsters given BBR intraperitoneally (20 mg/kg, single dose) was higher than those given BBR orally (100 mg/kg, single dose, n=6). **d-e** TMA/TMAO levels remained unchanged in plasma (**d**) and faeces (**e**) of the HFD-fed hamsters after intraperitoneal administration of BBR (20 mg/kg, n=6, *NS*: no significance). Data shown are mean ± SD and analysed with two-tailed student's-*t* test.

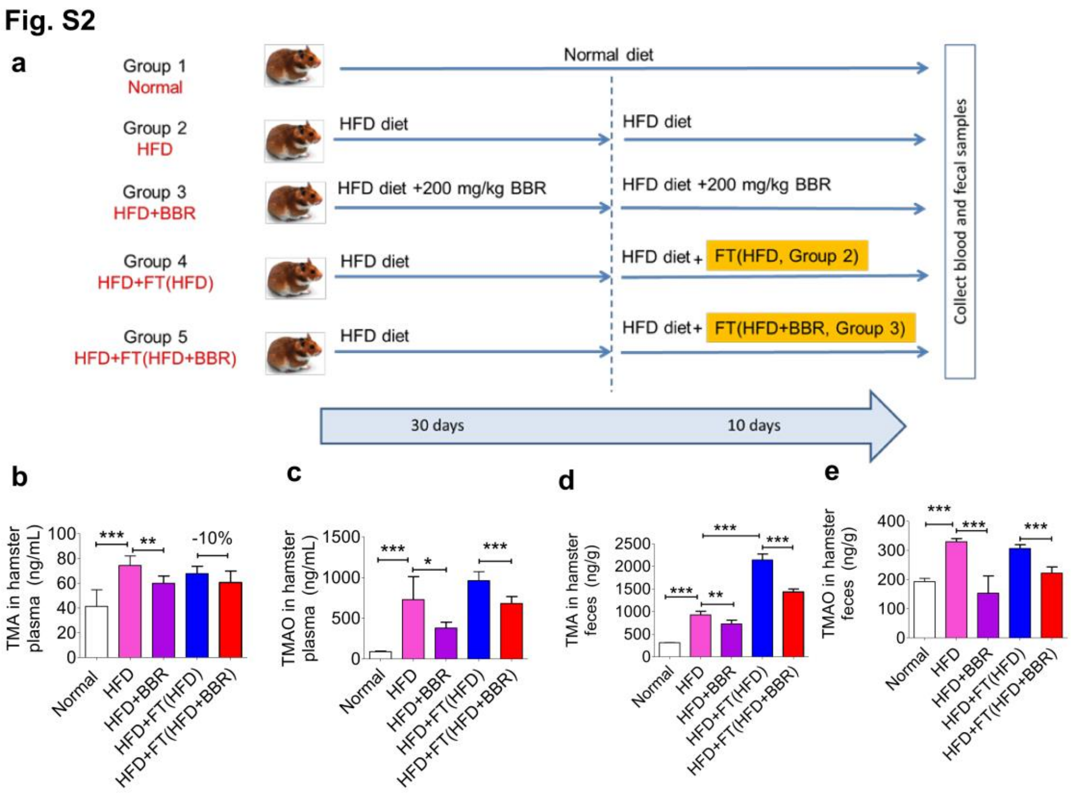

Fig. S2 The experiment of faecal transplantation (FT) in the high-fat diet (HFD)-fed hamsters.

a The schematic design of the fecal transplantation (FT) experiment of hamsters. **b-e** TMA and TMAO levels in plasma (**b, c**) and faeces (**d, e**) of HFD hamsters with fecal transplantation (FT). After fecal transplantation of BBR treated gut microbiota (FT-HFD+BBR), the levels of TMA and TMAO in plasma and faecal samples were significantly reduced by comparison with the fecal transplantation group of HFD treated group (FT-HFD). Normal, the normal control group; HFD, the high-fat diet group; HFD+BBR, the high-fat diet group treated with BBR (oral, 200 mg/kg); FT-HFD, the fecal transplantation group treated with fecal bacteria from HFD group; FT-HFD+BBR, the fecal transplantation group treated with fecal bacteria from HFD+BBR group (n=6). Data are expressed as mean ± SD and analyzed by two-tailed student's *t* test. *P<0.05, **P<0.01, ***P<0.001.

Fig. S3

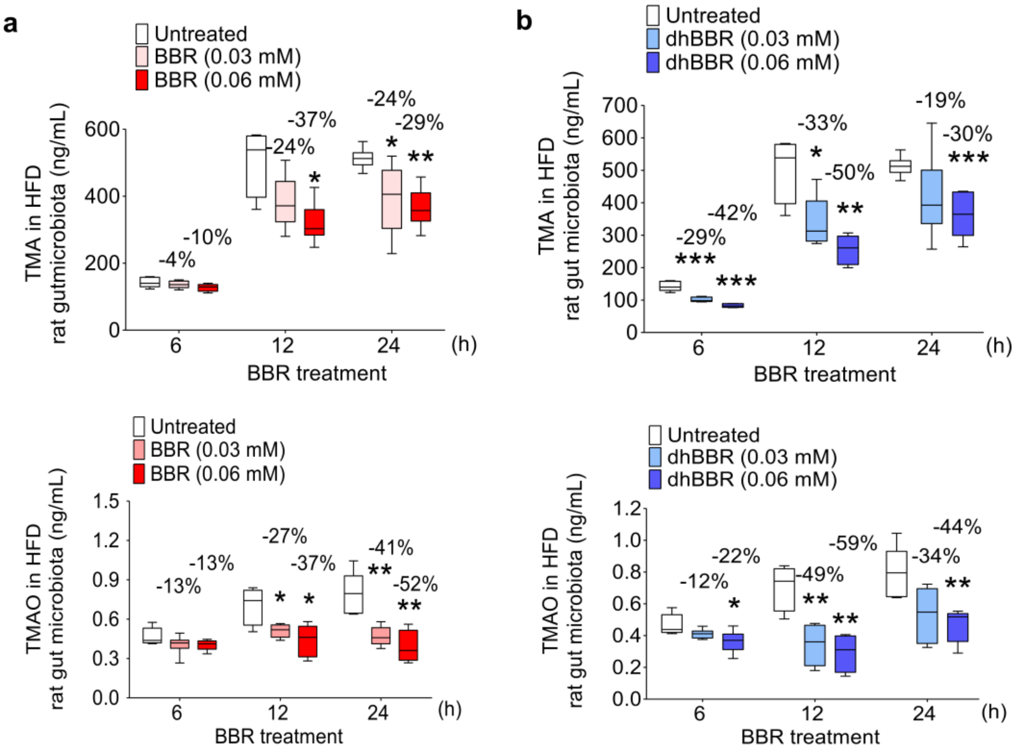

Fig. S3 BBR and dhBBR inhibited the production of TMA and TMAO in the intestinal bacteria from HFD-treated rat.

a TMA and TMAO levels in intestinal bacteria from the HFD-treated rats decreased significantly after BBR treatment (0.03, 0.06 mM) (n=6, *P<0.05 and **P<0.01). **b** DhBBR (0.03, 0.06 mM) *in vitro* significantly decreased the level of TMA and TMAO in the intestinal bacteria from HFD-treated rats (n=6, *P<0.05, **P<0.01 and ***P<0.001). Data shown are mean ± SD and analysed by two-tailed student's *t* test.

Fig. S4

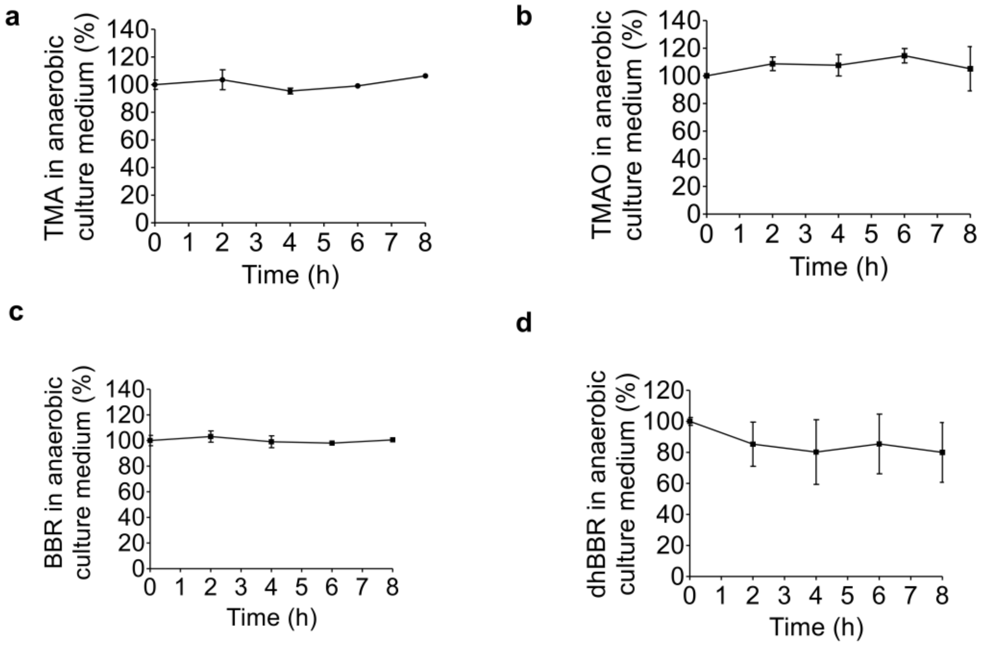

Fig. S4 The stability of TMA (**a**), TMAO (**b**), BBR (**c**) and dhBBR (**d**) in the anaerobic culture medium at 37 ℃ for 8 h (n=3). TMA, TMAO, BBR and dhBBR were basically stable in the bacterial culture medium under anaerobic condition for 8 h.

Fig. S5

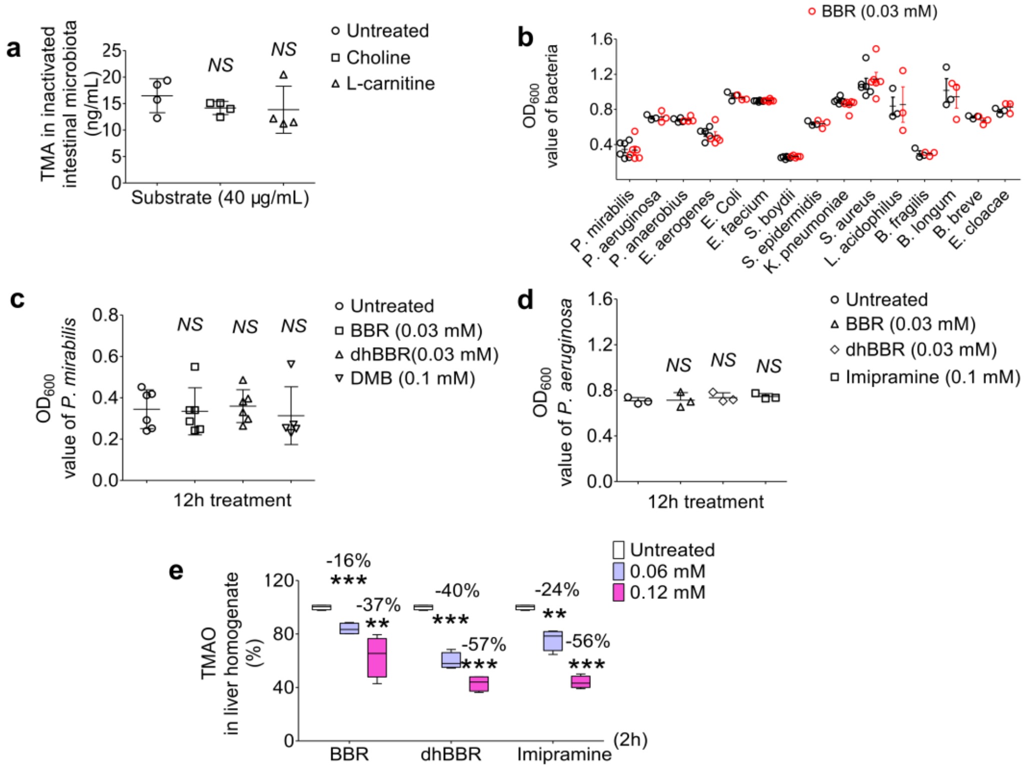

Fig. S5 Levels of TMA or TMAO and bacterial colony numbers after BBR/dhBBR treatment.

a TMA level remained unchanged in the heat-inactivated intestinal bacteria after adding identical concentration of choline or L-carnitine (40 μg/mL, n=4; *NS*: no significance). **b** The growth of 15 bacterial strains was not influenced by BBR (0.03 mM), via measuring the OD_{600nm} value. **c** The colony number of *P. mirabilis* was not influenced by BBR, dhBBR, or DMB, n=6. **d** The colony number of *P. aeruginosa* was not changed after treatment, n=3. **e** BBR, dhBBR and imipramine (0.06, 0.12 mM) inhibited TMAO production in live homogenate after 2h incubation (n=4, ***P*<0.01, ****P*<0.001). Data are expressed as the mean ± SD and analysed with two- tailed student's *t* test.

Fig. S6

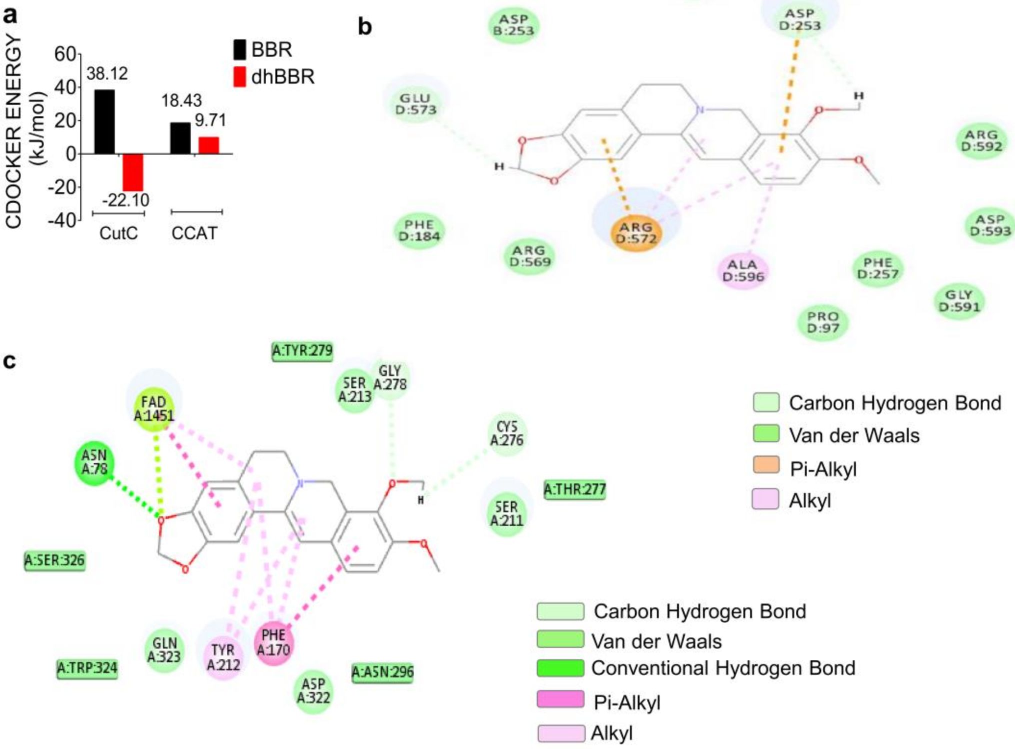

Fig. S6 Virtual docking results and interaction between dhBBR and CutC or FMO. **a** Virtual docking of BBR or dhBBR with bacterial choline-TMA lyase (CutC)/ carnitine coenzyme A transferase (CCAT). **b** Interaction between dhBBR and CutC in amino acids base. **c** Interaction between dhBBR and FMO in amino acids base.

Fig. S7

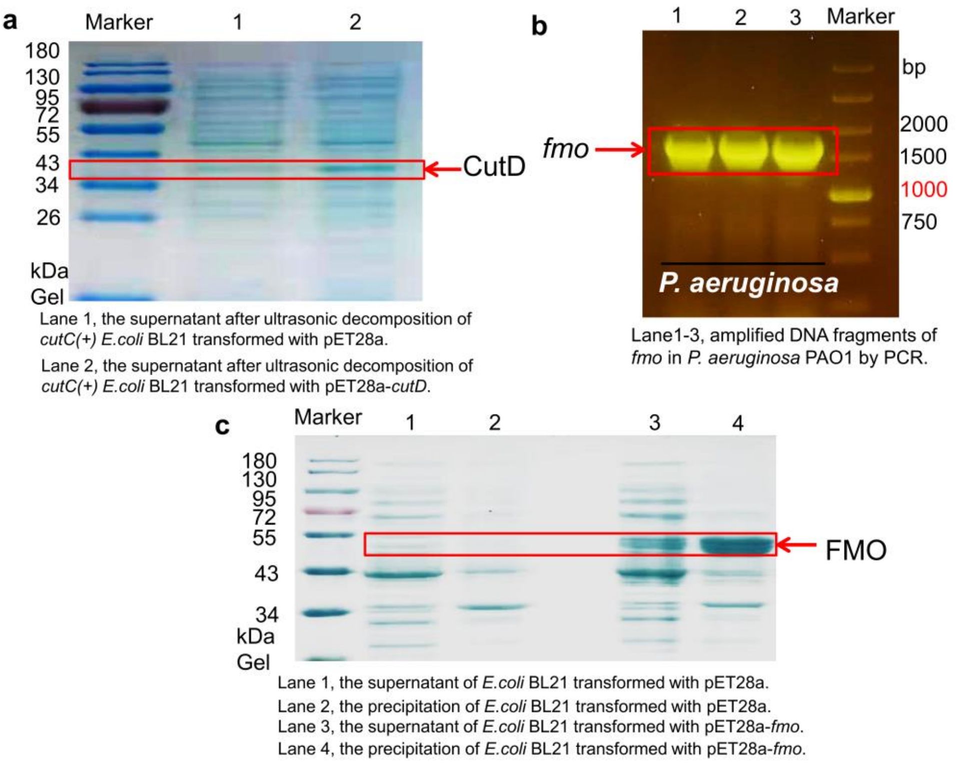

Fig. S7 The gel images of protein CutC/CutD or FMO and genes (*fmo*).

a CutD protein was expressed in the transformed *E. coli* BL21 cells. Lane 1, the supernatant of *cutC (+) E.coli* BL21 transformed with pET28a. Lane 2, the supernatant of *cutC (+) E.coli* BL21 transformed *with* pET28a-*cutD*. **b** Agarose gel electrophoresis of the *fmo* gene in *E. aeruginosa* PAO1 strains. Lane 1-3, amplified DNA fragments of the *fmo* in *P. aeruginosa* PAO1 through PCR amplification. **c** SDS-PAGE results showed that the protein FMO from *P. aeruginosa* PAO1 was expressed in *E. coli* BL21; the BL21 cells with pET28a plasmid was used as control. Lane 1, the supernatant of *E.coli* BL21 transformed with pET28a. Lane 2, the precipitation of *E.coli* BL21 transformed with pET28a. Lane 3, the supernatant of *E.coli* BL21 transformed with pET28a-*fmo*. Lane 4, the precipitation of *E.coli* BL21 transformed with pET28a-*fmo*.

Fig. S8

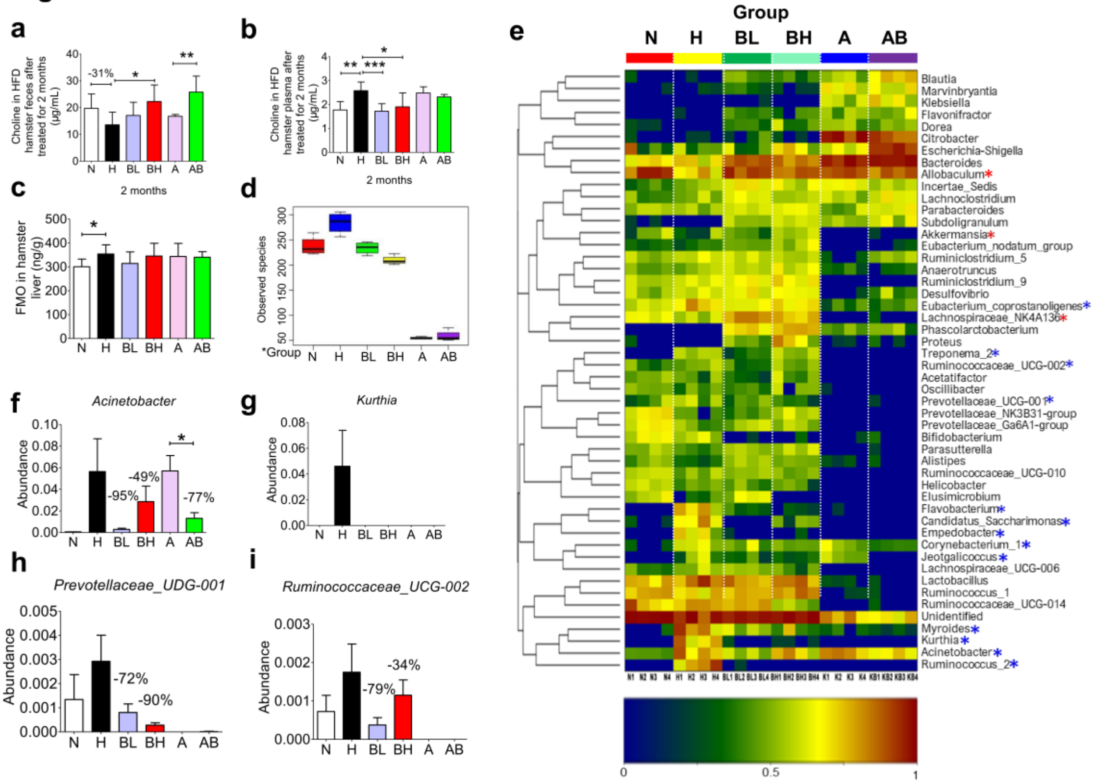

Fig. S8 BBR alleviated the symptoms of atherosclerosis in HFD-induced atherosclerotic hamsters.

a, b Choline levels in faeces (**a**) and plasma (**b**) samples of atherosclerotic hamsters after BBR treatment at 2 months. The consumption of choline in gut microbiota increased in the atherosclerosis model group, and oral BBR treatment significantly decreased the use of choline by bacteria and thereby increased the remaining of choline in all the BBR treatment groups. **c** FMO level in atherosclerotic hamster liver. FMO levels in atherosclerotic hamsters were higher than the normal control group; BBR treatment or antibiotics treatment did not significantly influence the FMO level in liver. **d** The observed bacterial species in hamster faeces in the study groups. Oral administration of berberine and combined antibiotics decreased the bacterial species. **e** The heat-map of the top 50 bacterial genera that exhibited the most substantial change in abundance after BBR treatment (the increased bacteria labeled in red * and the decreased in blue*). **f-i** BBR significantly decreased the abundance of TMA produced genera in the atherosclerotic hamster feces, including (**f**) *Acinetobacter*, (**g**) *Kurthia*, (**h**) *Prevotellaceae_UDG-001 and* (**i**) *Ruminococcaceae_UCG-002* (n=4). N, the normal hamster as control, n=8; H, the HFD fed-atherosclerotic hamster, n=7; BL, the HFD fed-atherosclerotic hamster treated with oral BBR (100 mg/kg/d), n=7; BH, the HFD fed-atherosclerotic hamster treated with oral BBR (200 mg/kg/d), n=4; A, the HFD fed-atherosclerotic hamster treated with antibiotics, n=5; AB, the HFD fed-atherosclerotic hamster treated with BBR (200 mg/kg/d) and the antibiotics, n=5. Data in **a-c** are expressed as mean ± SD and analyzed by two-tailed student's *t* test (**P*<0.05, ***P*<0.01, and ****P*<0.001), data in **f-i** are expressed as mean ± SEM.

Fig. S9

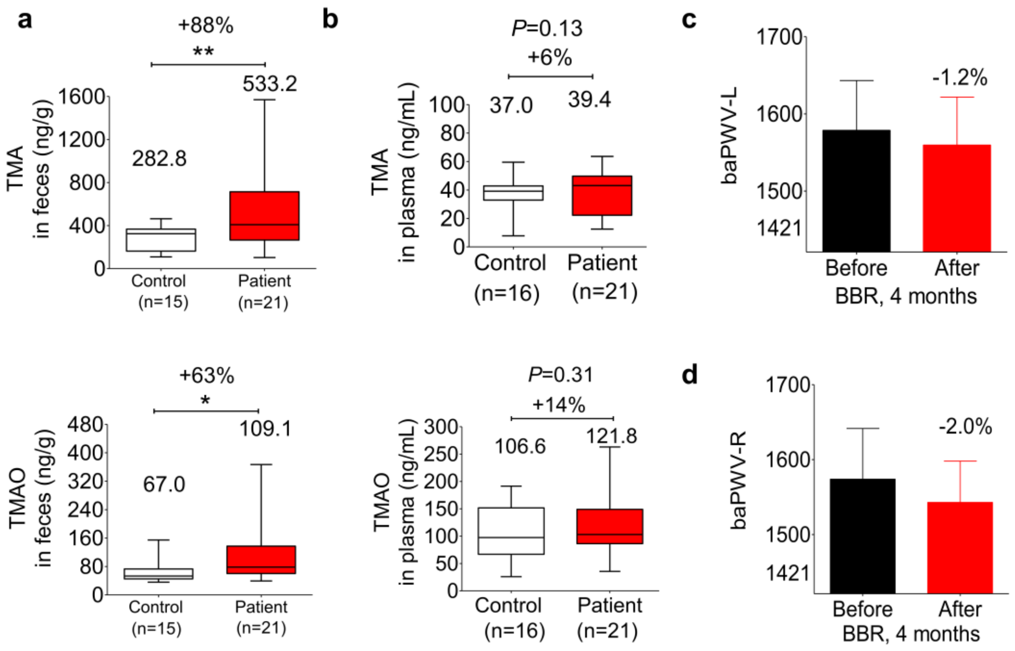

Fig. S9 Levels of TMA and TMAO in patients and BBR reduced the level of baPWV (brachial-ankle pulse wave velocity) in patients.

a Levels of TMA and TMAO in faeces of atherosclerosis patients were significantly higher than that in the control volunteers (+88%, $^{**}P<0.01$; +63%, $^{*}P<0.05$; 21 patients vs 15 volunteers). **b** TMA and TMAO levels in plasma of atherosclerosis patients seemed higher than that in the healthy volunteers (+6%, $P=0.13$; +14%, $P=0.31$; 21 patients *vs* 16 volunteers). **c** Left baPWV of atherosclerotic patients was decreased by 1.2 % after BBR treatment for 4 months. **d** Right baPWV of patients was decreased by 2.0 % after BBR treatment for 4 months. Data shown are mean ± SEM and analysed by one-tailed paired *t* test.

Fig. S10

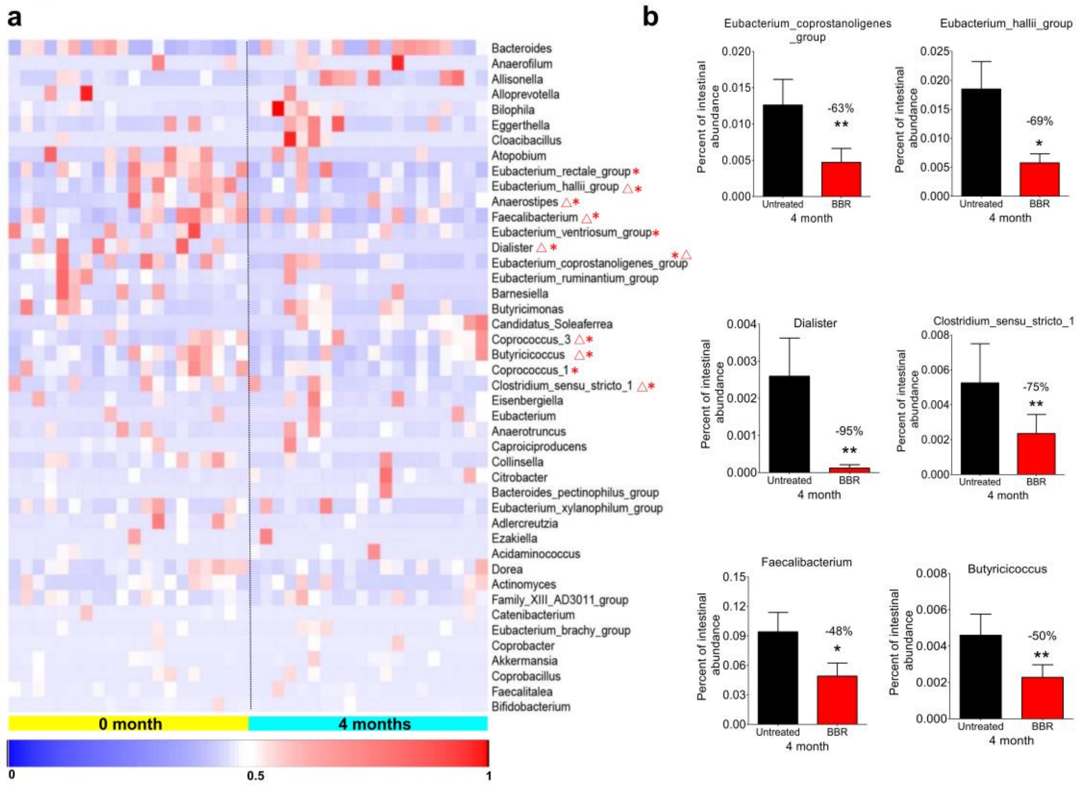

Fig. S10 Intestinal bacterial composition in patients after BBR treatment.

a The heat map of the top 50 bacterial genera that exhibited the most substantial change in abundance after BBR treatment in patients with atherosclerosis (the bacteria that decreased in abundance were labelled in red "*"). Abundance of *Eubacterium_hallii_group*, *Anaerostipes*, *Faecalibacterium*, *Dialister*, *Eubacterium_coprostanoligenes_group*, *Coprococcus_3*, *Butyricicoccus* and *Clostridium_sensu_strito_1* in gut microbiota which might produce TMA, decreased after treating with BBR for 4 months (labelled with red "Δ"). **b** The abundance of TMA-producing bacteria decreased. Among them, *Eubacterium_coprostanoligenes* decreased by 63% (**P<0.01), *Eubacteriu_hallii_group* decreased by 69% (*P<0.05), *Dialister* decreased by 95% (**P<0.01), *Clostridium_sersu_stricto_1* decreased by 75% (**P<0.01), *Faecalibacterium* decreased by 48% (*P<0.05), *Butyricicoccus* decreased by 50% (**P<0.01), *Anaerostipes* decreased by 32% and *Coprococcus_3* decreased by 24%. Data are expressed as the mean ± SEM and analysed by one-tailed paired *t* test.

Table S1 Coefficient of variation (CV) of TMA/TMAO by two methods

	Sample	Measured value by published method [42] (d9-TMA and d9-TMAO as the internal standards)	Measured value by this method (benzylamine as internal standard)	*CV (%)
TMA level (ng/mL)	Plasma-1	24.21	24.01	0.83
	Plasma-2	19.40	17.28	11.56
	Plasma-3	21.42	22.42	4.56
	Feces-1	208.16	224.55	7.58
	Feces-2	198.46	219.17	9.92
	Feces-3	215.87	220.42	2.09
TMAO level (ng/mL)	Plasma-1	103.18	99.45	3.68
	Plasma-2	98.82	87.44	12.22
	Plasma-3	132.53	122.91	7.53
	Feces-1	633.13	627.37	0.91
	Feces-2	225.64	197.37	13.37
	Feces-3	414.33	392.51	5.41

*CV: Coefficient of variation; CV (%) = (SD/Mean) × 100%

Table S2 Coefficient of variation (CV) of TMA/TMAO before and after treated with choline/carnitine

	Samples	Before choline added	After choline added (1 μg/mL)	*CV (%)
TMA level (ng/mL)	Plasma-1	23.56	24.74	4.89
	Plasma-2	27.18	29.89	9.50
	Plasma-3	14.07	14.20	0.92
	Feces-1	204.53	204.05	0.23
	Feces-2	190.32	192.21	0.99
	Feces-3	196.15	187.71	4.40
TMAO level (ng/mL)	Plasma-1	61.03	65.86	7.61
	Plasma-2	68.92	70.62	2.44
	Plasma-3	68.85	63.61	7.91
	Feces-1	171.34	164.35	4.16
	Feces-2	101.95	100.86	1.07
	Feces-3	252.13	248.50	1.45
		Before carnitine added	After carnitine added (1 μg/mL)	*CV (%)
TMA level (ng/mL)	Plasma-1	126.99	120.32	5.39
	Plasma-2	124.21	121.40	2.29
	Plasma-3	104.85	107.43	2.43
	Feces-1	219.43	220.67	0.56
	Feces-2	183.54	208.39	12.68
	Feces-3	200.48	217.48	8.13
TMAO level (ng/mL)	Plasma-1	18.77	20.36	8.13
	Plasma-2	19.59	19.77	0.91
	Plasma-3	18.77	16.59	12.33
	Feces-1	378.44	372.22	1.66
	Feces-2	180.96	199.90	9.95
	Feces-3	548.01	536.36	2.15

*CV: Coefficient of variation; CV (%) = (SD/Mean) × 100%

Table S3 Method sensitivity and linearity range

	TMA	TMAO
Instrumental LLOQ (ng/mL)	0.1	0.1
Instrumental LLOD (ng/mL)	0.05	0.05
Linear range (ng/mL)	0.2-500	0.2-500
R^2	0.9927	0.9932
Coefficient of variation in matrix effect (%, n=6)		
Plasma	1.17	0.72
Feces	0.33	0.40
Medium	5.98	3.23

Table S4 Accuracy and precision

		TMA			TMAO		
		**Added (ng/mL)**	**Accuracy (%)**	**RSD (%)**	**Added (ng/mL)**	**Accuracy (%)**	**RSD (%)**
Medium	Batch-1	5	99.30	8.00	2	98.92	3.05
		50	100.16	5.52	50	98.42	7.46
		400	102.71	4.84	400	100.74	3.28
	Batch-2	5	106.58	3.12	2	101.40	9.35
		50	99.97	3.84	50	100.62	7.73
		400	107.64	3.14	400	95.30	14.46
	Batch-3	5	95.22	2.39	2	99.17	3.62
		50	102.26	3.25	50	107.22	5.88
		400	94.84	2.46	400	106.56	2.67
	Inter-day	5	95.60	5.16	2	99.83	7.58
		50	100.91	10.30	50	102.09	8.12
		400	101.96	6.00	400	100.87	5.64
Feces	Batch-1	50	98.75	1.51	20	97.75	2.46
		100	99.22	1.28	100	106.23	1.79
		400	98.11	1.75	400	99.01	3.33
	Batch-2	50	108.26	1.08	20	103.30	4.13
		100	105.37	1.48	100	100.31	3.55
		400	99.36	1.91	400	109.88	5.01
	Batch-3	50	94.91	5.47	20	86.36	3.98
		100	106.61	2.73	100	109.4	2.46
		400	89.91	5.47	400	98.48	2.66
	Inter-day	50	100.64	5.69	20	95.80	3.87
		100	103.73	4.30	100	105.31	3.80
		400	95.79	4.68	400	102.46	4.40
Plasma	Batch-1	5	103.86	5.22	20	99.51	6.03
		100	101.56	5.59	100	104.18	2.65
		400	106.15	7.24	400	99.25	1.22
	Batch-2	5	99.91	6.62	20	98.42	7.64
		100	102.20	5.73	100	99.10	2.60
		400	109.3	2.31	400	97.59	2.70

Batch-3	5	100.44	2.28	20	107.05	9.48
	100	109.32	3.07	100	103.46	3.74
	400	104.38	2.29	400	100.49	4.51
Inter-day	5	101.40	9.40	20	101.66	8.73
	100	104.36	5.86	100	102.25	3.78
	400	106.61	5.02	400	99.11	3.33

Table S5 Extraction recovery

	TMA			TMAO		
	**Added (ng/mL)**	**Recovery (%)**	**RSD (%)**	**Added (ng/mL)**	**Recovery (%)**	**RSD (%)**
Medium	5	104.55	2.53	2	102.28	10.61
	50	101.95	2.31	50	101.63	4.44
	400	102.33	2.92	400	99.46	3.6
Feces	50	99.73	3.98	20	104.94	3.75
	100	97.51	2.32	100	94.27	3.02
	400	97.81	2.25	400	94.42	3.78
Plasma	5	96.31	5.89	20	101.49	3.97
	100	96.49	6.04	100	98.97	2.34
	400	87.47	3.17	400	98.2	2.34

Table S6 Matrix effects (recovery, n=10)

	TMA			TMAO		
	Added (ng/mL)	Recovery (%)	RSD (%)	Added (ng/mL)	Recovery (%)	RSD (%)
Plasma	100	105.72	8.57	100	99.71	2.75
	400	106.34	3.87	400	87.3	3.49
Feces	100	94.21	2.98	100	106.87	4.35
	400	108.01	5.56	400	97.12	9.28
Medium	100	106.04	8.08	100	96.63	8.35
	400	96.59	5.44	400	96.87	7.07

Table S7 Stability after treatment 24 h at 4 ℃

	TMA			TMAO		
	**Added (ng/mL)**	**Recovery (%)**	**RSD (%)**	**Added (ng/mL)**	**Recovery (%)**	**RSD (%)**
Medium	5	101.36	3.56	2	98.41	9.09
	50	105.7	3.42	50	93.83	2.72
	400	105.49	6.76	400	93.73	3.4
Feces	50	98.45	2.66	20	95.91	5.54
	100	102.03	2.8	100	108.1	1.75
	400	102.29	3.03	400	97.92	3.98
Plasma	5	99.9	2.74	20	100.58	2.87
	100	105.28	3.6	100	99.26	2.15
	400	103.14	3.11	400	101.78	1.16

Table S8 Baseline characteristics of the study cohorts

Characteristic*	Group 1 Non-atherosclerotic control (N=16)	Group 2 Berberine treatment group (N=21)	Group 3 Statin group (with anticoagulants, N=12)
Age-yr	60.50±8.89	63.67±5.23	55.60±8.85
Female-no.(%)	9 (56.25)	9 (42.86)	2 (16.67)
Body mass index†-kg/m^2	-	25.83±2.70	-
TC-mmol/L	4.59±0.57	5.70±1.04	4.53±1.10
TG-mmol/L	1.12±0.37	3.65±4.55	1.54±0.51
HDL-C-mmol/L	1.62±0.41	1.30±0.32	1.09±0.26
LDL-C-mmol/L	2.49±0.38	3.11±0.85	2.77±0.73
Glucose-mmol/L	5.16±0.49	6.79±2.29	7.71±3.44
Uric acid-umol/L	313±83	349±63.84	363±82
Urea-mmol/L	-	9.24±8.57	6.51±2.28
Creatinine-umol/L	-	74.19±18.33	67.20±13.54
Plaque score‡-mm	-	6.57±7.11	8.98±5.61
Medication§-no.(%)<br>Lipid-lowering agent	0 (0)	0 (0)	0 (0)

* Values presented are means ±SD.

Participants in non-atherosclerotic control group were diagnosed with no atherosclerosis by Doppler ultrasonography and did not received any drug treatment.

Patients in BBR treatment or statin group (with anticoagulants) were diagnosed with atherosclerosis using Doppler ultrasonography before enrolled in the study.

Abbreviation: TC, total cholesterol; TG, triglyceride; HDL-C, high density lipoprotein cholesterol; LDL-C, low density lipoprotein cholesterin; AST, aspartate aminotransferase; ALT, alanine aminotransferase;

† The body mass index is calculated by the value of weight in kilograms divided by the square of the height in meters.

‡ Plaque score is calculated by the sum of the thickness of the plaques per patient by Doppler ultrasonography at bilateral common carotid artery, bilateral carotid bifurcation, bilateral internal carotid artery, bilateral external carotid artery, bilateral vertebral artery intervertebral space and bilateral subclavian artery.

§The number of patients that have taken medicine including lipid-lowering agent in one month before enrollment.

Table S9 BBR improved biomarker profiles in the clinical cohort (n=21)

Indexes	Before BBR	After BBR
FBG (mmol/L)	6.79±2.29	6.39±2.39
TC (mmol/L)	5.70±1.04	5.24±0.83**#
TG (mmol/L)	3.65±4.55	2.73±4.00*
LDL-c (mmol/L)	3.11±0.85	2.99±0.75#
TMA in blood (ng/mL)	39.37±15.78	24.76±4.66***
TMAO in blood (ng/mL)	121.84±57.49	79.53±43.57*
TMA in feces (ng/mL)	533.18±362.49	332.38±119.56*
TMAO in feces (ng/mL)	109.05±79.67	77.10±47.54*

Data are expressed as the means ± SD.

*P<0.05, **P<0.01, ***P<0.001.

#The average values of the biomarkers returned to normal level.

Treatment: BBR hydrochloride, oral, 0.5 g×2/d, 4 months.

Table S10 Plaque size of the patients before and after BBR treatment

Plaque No*.	Participant No.	Code	Dist A before treatment (mm)	Dist B before treatment (mm)	Dist A after BBR treatment (mm)	Dist B after BBR treatment (mm)
1	1	Y GY	1.6	9.2	1.5	8
2	2	G DW	3.2	27.2	3.2	29.5
3	2	G DW	1.6	24.2	1.6	23.9
4	2	G DW	3.6	13.4	3.2	10.9
5	2	G DW	2.4	9	2.3	9.9
6	2	G DW	4	14.8	3.8	14.9
7	3	L JM	1.5	10.2	1.5	10.8
8	3	L JM	2.2	14	2.5	14.7
9	3	L JM	2.2	10.9	2	10.7
10	4	L ZF	2.3	8.8	2.6	8.7
11	4	L ZF	2.5	9.4	2.3	9.1
12	5	C K	1.4	7.6	1.5	6.3
13	5	C K	2.4	9.7	2.6	7.5
14	5	C K	3.8	15.3	3	14.2
15	6	T WM	2.3	5.1	2.4	6.3
16	6	T WM	2.9	10.2	2.3	10
17	6	T WM	2.8	11	2.4	9.9
18	6	T WM	2.3	6.7	2.3	6.5
19	6	T WM	1.5	11.3	1.8	7.1
20	6	T WM	4.7	9.4	4.6	9
21	7	W YH	2	5.2	2	5.4
22	8	L KJ	4.3	12.9	4.1	12.2
23	9	K LH	1.5	11.3	1.5	12.9
24	10	Z LY	2	8.1	1.6	8.2
25	11	L CX	2.9	9.6	2.8	9.6
26	12	F XL	2.2	5.1	2.2	5.1
27	12	F XL	2	6.8	1.9	6.9
28	13	L R	1.5	5	1.7	5.5
29	14	W SC	2.8	6.2	3.2	7
30	15	C JR	3.6	28.7	3.1	18.5
31	15	C JR	2.3	11.1	2.2	13
32	15	C JR	3.6	32.6	3.4	31.2
33	15	C JR	2.1	12.8	3.7	15
34	15	C JR	4.5	17.4	4	16.4
35	15	C JR	2.9	20.6	2.5	25.6
36	15	C JR	3.4	14.4	3.4	14.4

37	15	C JR	9.2	28.9	8.5	26.8
38	16	C LJ	2.2	8.4	1.9	6.7
39	17	Y XY	2.2	9.2	2	11.7
40	17	Y XY	5.7	21	6.1	21.9
41	18	S HB	1.7	8.8	1.9	7.9
42	18	S HB	3.2	10	3.5	13.9
43	19	Y CR	2.3	7.6	1.8	5.9
44	19	Y CR	4.2	18.8	2.9	13.5
45	20	S LT	2.2	9.7	2.2	7.9
46	21	Q SF	2	8.1	1.8	10
47	21	Q SF	2.5	7.7	2.8	9.6
48	21	Q SF	3.1	8.2	2.8	8.8
49	21	Q SF	2.6	12.1	2.6	11.1

*One patient might have several plaques.

Dist A: Carotid intima-media thickness.

Dist B: Carotid plaque length.

1 **Table S11 Plaque size of the patients before and after statin treatment**

Plaque No*.	Participant No.	Code	Dist A before treatment (mm)	Dist B before treatment (mm)	Dist A after statin treatment (mm)	Dist B after statin treatment (mm)
1	1	W JT	1.6	8.6	1.5	7.2
2	1	W JT	2.5	9.7	2.4	8.5
3	1	W JT	2	6.3	1.9	6.3
4	2	Z WT	2.8	7.8	2.6	7.7
5	2	Z WT	2.3	9.4	1.5	6.2
6	2	Z WT	2.3	11.5	2.4	11
7	2	Z WT	2.2	3.9	0	0
8	2	Z WT	1.6	3.7	0	0
9	2	Z WT	3.1	11.8	2.9	11.7
10	3	H Z	3.3	34.1	2.9	28.2
11	3	H Z	0	0	2.9	18
12	3	H Z	0	0	3.4	15.9
13	3	H Z	3.7	22.1	3.6	25.6
14	3	H Z	4.2	19.4	2.6	16.6
15	3	H Z	3.2	7.9	3.4	11.8
16	4	L SQ	1.9	5.6	1.7	5.6
17	4	L SQ	3.1	16.5	3	15.4
18	5	Z YL	2.2	14.9	2.1	14.7
19	5	Z YL	1.9	10.3	1.7	10.1
20	5	Z YL	2	7.4	1.9	7.3
21	5	Z YL	2.2	16.9	2.1	14.9
22	6	L SH	2.3	7.1	2.2	7
23	6	L SH	1.9	4.8	2.4	7.7
24	7	L W	2.8	10.5	3.5	11.8
25	8	S BJ	1.7	8.7	0	0
26	8	S BJ	0	0	3	13
27	8	S BJ	0	0	1.9	8.9
28	8	S BJ	1.8	9.9	2	9.1
29	8	S BJ	2.1	11.3	2.1	8.2
30	9	L JL	2.1	5.8	2	6.2
31	9	L JL	2.8	16.2	2.5	14.1
32	9	L JL	2.5	8.1	2.5	9.2
33	10	L CL	1.5	16.7	2.4	9
34	10	L CL	1.5	2.1	0	0
35	10	L CL	1.8	5.7	3.3	15.8
36	10	L CL	2.2	13.7	2.9	11.1

37	10	L CL	0	0	3.1	8.7
38	10	L CL	2.1	13.5	0	0
39	10	L CL	1.6	2.2	0	0
40	10	L CL	0	0	3.2	17.3
41	10	L CL	2	6.5	5.5	11.1
42	10	L CL	1.8	5.7	0	0
43	11	X ZQ	1.8	19.9	1.6	16.7
44	11	X ZQ	1.8	31.8	1.7	8.5
45	11	X ZQ	0	0	2.3	23
46	11	X ZQ	1.5	7.9	2.1	9.2
47	11	X ZQ	1.9	10.1	2	7.2
48	11	X ZQ	3.4	5.4	2.3	6.5
49	11	X ZQ	2.6	15.3	0	0
50	11	X ZQ	2	13.1	0	0
51	11	X ZQ	2.3	11.3	0	0
52	11	X ZQ	3.4	18.9	3.4	16.3
53	12	L ZE	2.1	5.7	1.2	6.4
54	12	L ZE	2.3	7.9	2.1	7.8

*One patient might have several plaques.

Dist A: Carotid intima-media thickness.

Dist B: Carotid plaque length.
